# Measuring Coverage in MNCH: A Prospective Validation Study in Pakistan and Bangladesh on Measuring Correct Treatment of Childhood Pneumonia

**DOI:** 10.1371/journal.pmed.1001422

**Published:** 2013-05-07

**Authors:** Tabish Hazir, Khadija Begum, Shams el Arifeen, Amira M. Khan, M. Hamidul Huque, Narjis Kazmi, Sushmita Roy, Saleem Abbasi, Qazi Sadeq-ur Rahman, Evropi Theodoratou, Mahmuda Shayema Khorshed, Kazi Mizanur Rahman, Sanwarul Bari, M. Mahfuzul Islam Kaiser, Samir K. Saha, A. S. M. Nawshad Uddin Ahmed, Igor Rudan, Jennifer Bryce, Shamim Ahmad Qazi, Harry Campbell

**Affiliations:** 1 Children's Hospital, Pakistan Institute of Medical Sciences, Islamabad, Pakistan; 2 International Centre for Diarrhoeal Disease Research, Bangladesh, Dhaka, Bangladesh; 3 Centre for Population Health Sciences, University of Edinburgh, Edinburgh, United Kingdom; 4 Dhaka Shishu (Children) Hospital, Dhaka, Bangladesh; 5 Department of International Health, Johns Hopkins Bloomberg School of Public Health, Johns Hopkins University, Baltimore, Maryland, United States of America; 6 Department of Maternal, Newborn, Child and Adolescent Health, World Health Organization, Geneva, Switzerland; Wellcome Trust Senior Research Fellow in Clinical Science, UCL Reader in International Child Health, Honorary Consultant, Great Ormond Street Hospital for Children, United Kingdom

## Abstract

**Background:**

Antibiotic treatment for pneumonia as measured by Demographic and Health Surveys (DHS) and Multiple Indicator Cluster Surveys (MICS) is a key indicator for tracking progress in achieving Millennium Development Goal 4. Concerns about the validity of this indicator led us to perform an evaluation in urban and rural settings in Pakistan and Bangladesh.

**Methods and Findings:**

Caregivers of 950 children under 5 y with pneumonia and 980 with “no pneumonia” were identified in urban and rural settings and allocated for DHS/MICS questions 2 or 4 wk later. Study physicians assigned a diagnosis of pneumonia as reference standard; the predictive ability of DHS/MICS questions and additional measurement tools to identify pneumonia versus non-pneumonia cases was evaluated.

Results at both sites showed suboptimal discriminative power, with no difference between 2- or 4-wk recall. Individual patterns of sensitivity and specificity varied substantially across study sites (sensitivity 66.9% and 45.5%, and specificity 68.8% and 69.5%, for DHS in Pakistan and Bangladesh, respectively). Prescribed antibiotics for pneumonia were correctly recalled by about two-thirds of caregivers using DHS questions, increasing to 72% and 82% in Pakistan and Bangladesh, respectively, using a drug chart and detailed enquiry.

**Conclusions:**

Monitoring antibiotic treatment of pneumonia is essential for national and global programs. Current (DHS/MICS questions) and proposed new (video and pneumonia score) methods of identifying pneumonia based on maternal recall discriminate poorly between pneumonia and children with cough. Furthermore, these methods have a low yield to identify children who have true pneumonia. Reported antibiotic treatment rates among these children are therefore not a valid proxy indicator of pneumonia treatment rates. These results have important implications for program monitoring and suggest that data in its current format from DHS/MICS surveys should not be used for the purpose of monitoring antibiotic treatment rates in children with pneumonia at the present time.

*Please see later in the article for the Editors' Summary*

## Editors' Summary

**Background:**

Pneumonia is a major cause of death in children younger than five years across the globe, with approximately 1.2 million children younger than five years dying from pneumonia every year. Pneumonia can be caused by bacteria, fungi, or viruses. It is possible to effectively treat bacterial pneumonia with appropriate antibiotics; however, only about 30% of children receive the antibiotic treatment they need. The Millennium Development Goals (MDGs) are eight international development goals that were established in 2000. The fourth goal (MDG 4) aims to reduce child mortality, specifically, to reduce the under-five mortality rate by two-thirds, between 1990 and 2015. Given that approximately 18% of all deaths in children under five are caused by pneumonia, providing universal coverage with effective treatments for pneumonia is an important part of MDG 4.

To ensure that MDG 4 targets are met, it is important to measure progress in providing effective treatments. For pneumonia, one of the key indicators for measuring progress is the proportion of children with pneumonia in a population who receive antibiotic treatment, also known as the antibiotic treatment rate. The antibiotic treatment rate is often measured using surveys, such as the Demographic and Health Surveys (DHS) and Multiple Indicator Cluster Surveys (MICS), which collect nationally representative data about populations and health in developing countries.

**Why Was This Study Done?:**

Concerns have been raised about whether information collected from DHS and MICS is able to accurately identify cases of pneumonia. In a clinical setting, pneumonia is typically diagnosed based on a combination of physical symptoms, including coughing, rapid breathing, or difficulty breathing, and a chest X-ray. The surveys rely on information collected from interviews of mothers and primary caregivers using structured questions about whether the child has experienced physical symptoms in the past two weeks and whether these were chest-related. The DHS survey labels this condition as “symptoms of acute respiratory infection,” while the MICS survey uses the term “suspected pneumonia.” Thus, these surveys provide a proxy measure for pneumonia that is limited by the reliance on the recall of symptoms by the mother or caregiver. Here the researchers have evaluated the use of these surveys to discriminate physician-diagnosed pneumonia and to provide accurate recall of antibiotic treatment in urban and rural settings in Pakistan and Bangladesh.

**What Did the Researchers Do and Find?:**

The researchers identified caregivers of 950 children under five years with pneumonia and 980 who had a cough or cold but did not have pneumonia from urban and rural settings in Pakistan and Bangladesh. Cases of pneumonia were identified based on a physician diagnosis using World Health Organization guidelines. They randomly assigned caregivers to be interviewed using DHS and MICS questions with either a two- or four-week recall period. They then assessed how well the DHS and MICS questions were able to accurately diagnose pneumonia and accurately recall antibiotic use. In addition, they asked caregivers to complete a pneumonia score questionnaire and showed them a video tool showing children with and without pneumonia, as well as a medication drug chart, to determine if these alternative measures improved the accuracy of pneumonia diagnosis or recall of antibiotic use. They found that both surveys, the pneumonia score, and the video tool had poor ability to discriminate between children with and without physician-diagnosed pneumonia, and there were no differences between using two- or four-week recall. The sensitivity (proportion of pneumonia cases that were correctly identified) ranged from 23% to 72%, and the specificity (the proportion of “no pneumonia” cases that were correctly identified) ranged from 53% to 83%, depending on the setting. They also observed that prescribed antibiotics for pneumonia were correctly recalled by about two-thirds of caregivers using DHS questions, and this increased to about three-quarters of caregivers when using a drug chart and detailed enquiry.

**What Do These Findings Mean?:**

The findings of this study suggest that the current use of questions from DHS and MICS based on mother or caregiver recall are not sufficient for accurately identifying pneumonia and antibiotic use in children. Because these surveys have poor ability to identify children who have true pneumonia, reported antibiotic treatment rates for children with pneumonia based on data from these surveys may not be accurate, and these surveys should not be used to monitor treatment rates. These findings should be interpreted cautiously, given the relatively high rate of loss to follow-up and delayed follow-up in some of the children and because some of the settings in this study may not be similar to other low-income settings.

**Additional Information:**

Please access these websites via the online version of this summary at http://dx.doi.org/10.1371/journal.pmed.1001422.

- More information is available on the United Nations goal to reduce child mortality (MDG 4)
- The World Health Organization provides information on pneumonia, its impact on children, and the global action plan for prevention and control of pneumonia
- More information is available on Demographic and Health Surveys and Multiple Indicator Cluster Surveys
- KidsHealth, a resource maintained by the Nemours Foundation (a not-for-profit organization for children's health) provides information for parents on pneumonia (in English and Spanish)
- MedlinePlus provides links to additional information on pneumonia (in English and Spanish)

*This paper is part of the* PLOS Medicine *“Measuring Coverage in MNCH” Collection.*

## Introduction

Globally, of the estimated 6.9 million annual deaths in children younger than 5 y, 1.2 million (18%) die from pneumonia [1]. Prompt treatment with appropriate antibiotics in children with pneumonia is an effective intervention for reducing mortality [2],[3]. The proportion of children with pneumonia in a population who receive antibiotic treatment (antibiotic treatment rate) is a key indicator for tracking progress in achieving Millennium Development Goal 4 targets [4]. The validity of this indicator depends on both the correct identification of pneumonia and the use of an antibiotic to treat the condition. Current measures of antibiotic use in pneumonia rely on household-based surveys such as the Demographic and Health Surveys (DHS) and the Multiple Indicator Cluster Surveys (MICS). It is, therefore, crucial that the DHS and MICS measures are reliable and accurate.

Pneumonia indicators in current surveys are based on interviews with mothers (DHS) or primary caregivers (MICS) using structured questions on cough and short, rapid breathing or difficulty in breathing in the previous 2 wk and whether these were chest-related. The intent is to identify the best possible proxy for pneumonia in order to assess treatment coverage based on mother/caregiver recall. Although the DHS survey labels this condition as “symptoms of acute respiratory infection,” the MICS survey uses the term “suspected pneumonia.” Irrespective of the terminology used, for program purposes these cases are classified as pneumonia. Therefore, the accuracy and reliability of these questions and the algorithm as a valid proxy of pneumonia in children has been questioned [5],[6], and there is a need to assess the validity of this approach.

Our primary study objective was to assess the validity of the caregiver's responses to the standard DHS/MICS questions about whether the child had pneumonia in the recent past, and if so, how it was treated. The reference standard was physician-diagnosed pneumonia (as per World Health Organization [WHO] definitions) [7]. A further objective was to determine if these measures can be improved by adding additional questions to the DHS/MICS surveys or using alternative measurement tools. We also aimed to assess if there was any difference in a caregiver's recall at 2 wk (recall period in current DHS/MICS surveys) and 4 wk.

## Methods

### Ethics Statement

Ethical approval was obtained from the Hospital Ethics Committee (Pakistan Institute of Medical Sciences), the Ethical Review Committee of the International Centre for Diarrhoeal Disease Research, Bangladesh, and the WHO Ethics Review Committee.

We employed a two-stage written consent procedure: caregivers were informed about the study and permission was obtained first at the time of diagnosis and enrollment, and then again at the start of the follow-up home interviews.

### Setting

DHS and MICS surveys are conducted very widely throughout low- and middle-income countries and in very diverse settings, which range from rural settings served by community services to urban and peri-urban settings, where the first level health services are provided by local hospital outpatient departments. We thus conducted the study in three diverse urban and rural settings.

The study was conducted in two countries: Pakistan and Bangladesh. In Pakistan, study participants were recruited in an urban setting, from the outpatient department of Children's Hospital, Pakistan Institute of Medical Sciences, Islamabad, Pakistan. In Bangladesh, the participants were recruited from both urban (Dhaka Shishu Hospital, a tertiary pediatric hospital) and rural (community-based recruitment in four unions [Mirzapur, Gorai, Bhatgram, and Jamurki] of Mirzapur, 63 km north of Dhaka) settings. The latter setting involved initial identification of pneumonia and “no pneumonia” cases during weekly home visits by Village Health Workers, later reassessed by study physicians according to WHO guidelines and enrolled.

### Study Design

The study identified and recruited two groups of children with acute respiratory infections: those who were confirmed to have pneumonia and those who did not have pneumonia. Two to four weeks after recruitment caregivers were surveyed to assess the accuracy of their recall of the diagnosis and treatment given. This enabled an assessment to be made of the degree to which DHS and MICS measures of antibiotic treatment in those with reported symptoms of pneumonia were valid measures of the antibiotic treatment of true pneumonia in a study population.

The study had two phases. Phase 1 involved recruiting large numbers of children with symptoms of acute respiratory infection and having trained study physicians establish whether pneumonia (the reference standard for the test) was present or absent. We purposefully selected hospital outpatient departments as two of the three study sites to increase the probability that we would find sufficient numbers of children presenting with symptoms of acute respiratory infection, and to allow careful training and monitoring of the study physicians to ensure that the reference standard was robust. At the third, rural site in Bangladesh, patients were also identified in the community. Phase 2 involved having trained field workers interview the caregiver of each child in their home using DHS and MICS algorithms and alternative tools, either 2 or 4 wk after their recruitment.

### Participants and Selection Criteria

The study participants were children 0–59 mo with physician-diagnosed clinical pneumonia or “no pneumonia” and their caregivers. A single WHO technical expert (S. A. Q.) oversaw staff training and study monitoring to ensure comparability of case definitions across sites.

### Enrollment and Follow-Up

#### Enrollment procedure

In the two urban settings (Islamabad and Dhaka) all children aged 0 to 5 y who were assessed and managed by hospital outpatient department physicians were referred to study physicians, who then screened, reassessed, and diagnosed the children using WHO acute respiratory infection guidelines and determined their eligibility for inclusion in the study. Children were enrolled in one of two groups: those with pneumonia and those with “no pneumonia” [7]. Treatment provided by outpatient department physicians was recorded. Children with the following conditions were excluded: recurrent wheezing (airway disorder/asthma), severe pneumonia requiring hospitalization (since DHS and MICS surveys typically gather data on very few of these cases due to their very low period prevalence in community surveys and since hospitalization, being a dramatic event, could induce recall bias), symptoms of chronic cough of more than 4 wk duration, history of pneumonia within past 10 d, history of congenital heart disease, and nonresident of study catchment area. Children with “cough or cold/no pneumonia” were frequency matched to pneumonia cases so that in any one week the age (and sex, in the case of the Bangladesh sites) distribution of cases of pneumonia and “cough or cold” was similar.

For each pneumonia case enrolled, a corresponding age-matched (and sex-matched, in case of Bangladesh sites) “no pneumonia” case was selected using a computer-generated randomization list. Details of the recruitment procedures for each site are given in Box 2.

**Box 1. Key DHS and MICS Acute Respiratory Infection Questions Used for the Identification of Children with a Caregiver Report of Symptoms and Signs of Pneumonia**

Q.535 (PDHS)/Q.B3 (BDHS)/Q.CA8 (MICS-P)/Q.C2 (MICS-B): When (name) had an illness with a cough, did s/he breathe faster than usual with short, rapid breaths or have difficulty in breathing?

Q.536 (PDHS)/Q.B4 (BDHS)/Q.CA9 (MICS-P)/Q.C3 (MICS-B): Was the fast or difficult breathing due to a problem in the chest or to a blocked or runny nose?

BDHS, Bangladesh DHS; MICS-B, MICS Bangladesh; MICS-P, MICS Pakistan; PDHS, Pakistan DHS.

**Box 2. Details of the Matching Procedures at the Study Sites**

**Pakistan**

For each pneumonia case enrolled a corresponding age-matched “no pneumonia” case was randomly selected. Randomization was done with a computer-generated randomization list with blocks of four (each having a randomly selected number). For every enrolled pneumonia case, a “no pneumonia” case was picked as per the sequence number of the corresponding block. There were two separate randomization lists: one for children 0–12 mo and one for those >12–59 mo (to match for age). Time matching was also ensured, whereby in any one week a similar number of age-matched “no pneumonia” and pneumonia cases were enrolled.

**Bangladesh**

The enrollment and randomization procedure followed a hierarchical procedure where an attempt was made to match each pneumonia case in a given week one-on-one with a non-pneumonia case based on age, sex, and study physician. The procedure for matching, in order of preference, was as follows:

1. Matched on case's sex and age (±2 mo), and assessed by the same study physician
2. Matched on case's sex and age (±2 mo), and assessed by different study physician
3. Matched on case's sex and same age category (≤12 mo or >12 mo), and assessed by the same study physician
4. If criteria 1–3 could not be met, we matched on case's sex and same age category under different study physician

If more than one child with no pneumonia was found for a case (pneumonia), we randomly selected only one control using computer-generated random numbers. A child was selected as a control only once. This procedure was followed at the end of each week throughout the enrollment period. An additional criterion was added after 2 mo of study enrollment to ensure adequate enrollment of controls: if matched controls were not found from the children with “no pneumonia” assessed in the same week as the cases, controls were selected from children with “no pneumonia” assessed in the week prior to or following the week of the case. Eligible and consented pneumonia patients were excluded from the study if no age- and sex-matched “no pneumonia” patient could be found.

In the rural setting (Mirzapur), the Village Health Workers identified all children with pneumonia or “no pneumonia” through their weekly household visits and informed the study physicians by mobile phone. The study physicians visited at home all children with “possible pneumonia” and a sample of children with “no pneumonia” who lived nearest to the case of “possible pneumonia.” They reassessed using Integrated Management of Childhood Illness guidelines, and screened and enrolled eligible children.

#### DHS and MICS questionnaires and alternative measurement tools

DHS and MICS surveys both have an algorithm of questions about the presence or absence of specific signs and symptoms of suspected pneumonia (denoted as “symptoms of acute respiratory infection” in the DHS survey and “suspected pneumonia” in the MICS questionnaire; copies of questionnaires are given in Texts S1, S2, S3). Additional study tools were developed by ARI Research Cell, Children's Hospital, Pakistan Institute of Medical Sciences, Islamabad, and field tested in both Pakistan and Bangladesh. These tools included a pneumonia score questionnaire, which consisted of questions on 20 commonly reported signs and symptoms of pneumonia (Box 3), and a video depicting children in three scenarios: with signs and symptoms of pneumonia, with severe pneumonia, and with cough and cold but without pneumonia. The video had nine different clips of no pneumonia, pneumonia (fast breathing), and severe pneumonia (lower chest indrawing), one set for each of three age groups, i.e., up to 2 mo, 2–11 mo, and 12–59 mo. Country-specific drug charts (flip chart and computer-based) were developed showing medicines (especially antibiotics) commonly used in pneumonia and other febrile illnesses in children 0–59 mo old. All questionnaires, including standard DHS and MICS questionnaires, were translated into local languages (Urdu and Bangla—see Texts S1, S2, S3 for copies of questionnaires).

**Box 3. Pneumonia Score Features**

1. Cough
2. Fever
3. Chills/sweats
4. Restlessness
5. Irritability
6. Loss of appetite
7. Abnormally sleepy
8. Wheezing
9. Shortness of breath
10. Fast breathing
11. Flaring of nostrils
12. Refusal to drink
13. Lower chest indrawing
14. Chest pain
15. Difficulty in breathing
16. Vomiting
17. Grunting
18. Blue coloration of skin
19. Coughing up blood
20. Convulsions

#### Home follow-up

Using computer-generated randomization lists, two-thirds of enrolled children were randomly allocated to follow-up at 2 wk, and one-third to follow-up at 4 wk. The mothers/caretakers were (i) interviewed using DHS/MICS questions on cough and chest-related short, rapid breathing and difficult breathing, (ii) asked about specific signs of pneumonia using a pneumonia score questionnaire, (iii) shown video clips to find which clip best represented their child's respiratory illness episode, and (iv) shown a “computer-based drug chart” and “a drug flip chart” to identify the drugs the child was treated with. The order of administering the DHS and MICS questionnaires was alternated with each child, so that each questionnaire was administered first on 50% of occasions.

### Data Management and Analysis

After double data entry and data cleaning, descriptive statistics were used to assess the socio-demographic and clinical characteristics of the study children. Sensitivity and specificity were calculated to assess the discriminative power of each measurement tool (DHS, MICS, pneumonia score questionnaire, pneumonia video) and for various combinations of tools.

For the pneumonia score, a composite score was developed by adding one point for each symptom or sign present. Sensitivity and specificity were calculated for each of the composite scores (ranging from lowest to highest). The intent was to identify a cutoff that can be used to reach a decision on the presence or absence of pneumonia. SPSS 11.0 (SPSS Inc.) and STATA 10 (StataCorp) were used in Pakistan and Bangladesh, respectively.

### Sample Size Calculation

Based on baseline estimates of sensitivity of 60%–70% and specificity of 70%–90% of mother/caregiver recall of symptoms of acute respiratory infection or suspected pneumonia in predicting true pneumonia, it was estimated that 300 children under 5 y with physician-diagnosed pneumonia and 300 with “no pneumonia” should be enrolled at each site in order to estimate sensitivity and specificity with a precision of ±5%.

The study is reported in line with the STARD statement (checklist in Text S4). The protocol is provided in Text S5.

## Results

### Enrollment and Follow-Up Status

**Figure 1:**
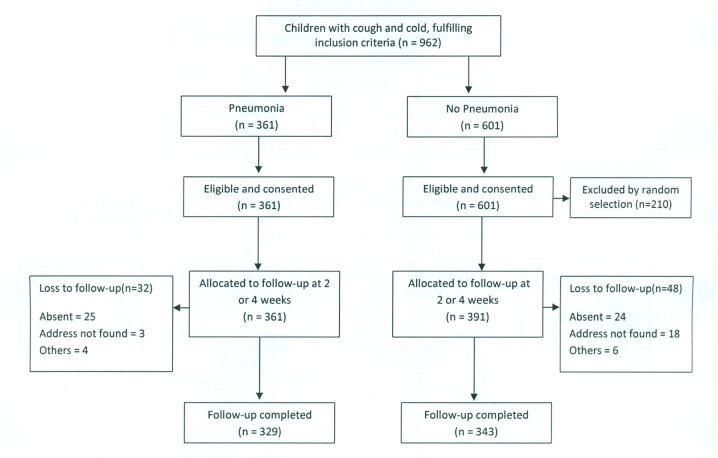
Pakistan urban site—flowchart of selection of pneumonia and no-pneumonia cases.

At the Pakistan site, 752 children were enrolled in the study—361 with pneumonia and 391 with “no pneumonia”—between October 2010 and February 2011 (Figure 1). Follow-up of 329 pneumonia and 343 “no pneumonia” cases was successfully carried out; 456 at 2 wk and 216 at 4 wk.

**Figure 2:**
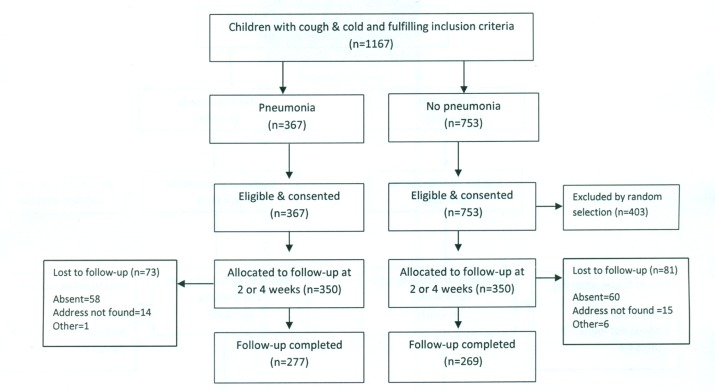
Bangladesh urban site—flowchart of selection of pneumonia and no-pneumonia cases in Dhaka Shishu Hospital, Bangladesh.

**Figure 3:**
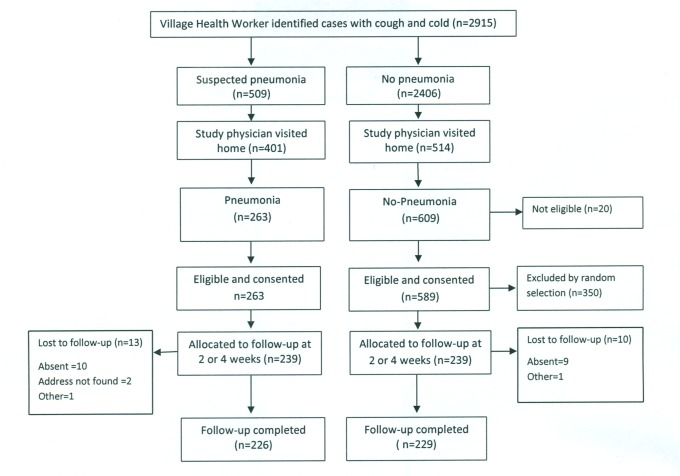
Bangladesh rural site—flowchart of selection of pneumonia and no-pneumonia cases in the rural setting.

In Bangladesh, between March and August 2011, 1,178 children—589 each in the pneumonia and “no pneumonia” groups—were enrolled, of which 700 were from Dhaka Shishu Hospital (Figure 2) and 478 from rural Mirzapur (Figure 3). “No pneumonia” cases were enrolled using recruitment procedures similar to those used in Pakistan.

### Baseline Characteristics of the Study Children

The socio-demographic characteristics and the clinical features of the study children are presented in Table 1.

**Table 1:** Baseline characteristics of the study children.

Characteristic	Pakistan (Urban)		Bangladesh (Urban)		Bangladesh (Rural)	
	Pneumonia (*n* = 329)	No Pneumonia (*n* = 343)	Pneumonia (*n* = 350)	No Pneumonia (*n* = 350)	Pneumonia (*n* = 239)	No Pneumonia (*n* = 239)
**Age of child (months) (mean ± SD)**	10.6±10.1	12.9±13.2	12.2±12.0	12.8±12.8	16.8±12.6	18.9±14.6
**Age category of child (months)**						
0 to 2	27 (8.2)	34 (9.9)	84 (24.0)	88 (25.1)	1 (0.4)	10 (4.2)
2 to 11	208 (63.2)	210 (61.2)	116 (33.1)	118 (33.7)	84 (35.2)	83 (34.7)
12 to 59	94 (28.6)	99 (28.9)	150 (42.9)	144 (41.1)	154 (64.4)	146 (61.1)
**Gender**						
Male	199 (60.5)	172 (50.1)	204 (58.4)	203 (58.1)	123 (51.5)	124 (51.9)
Female	130 (39.5)	171 (49.9)	146 (41.6)	147 (41.9)	116 (48.5)	115 (48.1)
**Siblings**						
No siblings	73 (22.2)	83 (24.2)	182 (52.0)	166 (47.4)	96 (40.2)	115 (48.1)
One or more	256 (77.8)	260 (75.8)	168 (48.0)	184 (52.6)	143 (59.8)	124 (51.9)
**Age category of mothers (years)**						
≤30	266 (80.9)	266 (77.6)	322 (92.0)	314 (89.7)	206 (86.2)	219 (91.6)
>30	63 (19.1)	77 (22.4)	28 (8.0)	36 (10.3)	33 (13.8)	20 (8.4)
**Mother's education**						
Illiterate	88 (26.7)	55 (16.0)	81 (23.1)	64 (18.3)	32 (13.4)	26 (10.9)
Up to 10th grade	145 (44.1)	157 (45.8)	192 (54.8)	209 (59.7)	175 (73.2)	176 (73.8)
Above 10th grade	96 (29.2)	131 (38.2)	77 (22.0)	77 (22.0)	32 (13.4)	38 (15.9)
**Father's education**						
Illiterate	42 (12.8)	18 (5.2)	58 (16.6)	52 (15.7)	47 (19.7)	29 (12.1)
Up to 10th grade	148 (45.0)	158 (46.1)	179 (51.1)	153 (43.7)	141 (59.0)	139 (58.1)
Above 10th grade	139 (42.2)	167 (48.7)	113 (32.3)	142 (40.6)	51 (21.4)	71 (29.7)
**Father's occupation status**						
Employed	322 (97.9)	322 (93.9)	343 (98.0)	345 (98.6)	226 (94.6)	231 (96.7)
Unemployed	7 (2.1)	21 (6.1)	7 (2.0)	5 (1.4)	13 (5.4)	8 (3.3)
**Symptoms**						
Cough and cold	327 (99.4)	343 (100.0)	342 (97.7)	343 (98.0)	228 (95.4)	237 (99.2)
Fever	296 (90.0)	192 (56.0)	153 (43.7)	100 (28.6)	77 (32.2)	42 (17.6)
Breathing problem	51 (15.5)	3 (0.9)	60 (17.1)	34 (9.7)	40 (16.7)	8 (3.4)
Feeding problem	16 (4.9)	0 (0.0)	0 (0.0)	0 (0.0)	0 (0.0)	0 (0.0)
Vomiting	10 (3.0)	2 (0.6)	0 (0.0)	0 (0.0)	0 (0.0)	0 (0.0)
Gastrointestinal problem	5 (1.5)	0 (0.0)	0 (0.0)	0 (0.0)	0 (0.0)	0 (0.0)
Irritability	5 (1.5)	0 (0.0)	0 (0.0)	0 (0.0)	0 (0.0)	0 (0.0)
Others	2 (0.6)	0 (0.0)	11 (3.1)	21 (6.1)	12 (5.0)	5 (2.0)
**Respiratory rate/minute (mean ± SD)**						
0–2 mo	70.5±11.3	46.8±8.8	63.8±4.4	43.4±5.4	61^a^	46.3±9.5
2–11 mo	64.5±10.1	37.2±6.0	55.1±5.1	37.9±5.9	54.7±5.5	37.0±7.2
12–59 mo	57.0±10.3	31.2±4.2	47.4±6.7	30.6±5.0	46.8±6.3	30.7±4.7
**Temperature (°C)**						
<37.5	236 (71.7)	309 (90.1)	216 (64.1)	293 (85.4)	201 (84.1)	228 (95.6)
≥37.5	93 (28.3)	34 (9.9)	121 (35.9)	50 (14.6)	38 (15.9)	11 (4.4)
**Findings on auscultation**						
No significant findings	120 (36.5)	334 (97.4)	194 (55.4)	320 (91.4)	190 (79.5)	233 (97.5)
Crepitations/wheeze	209 (63.5)	9 (2.6)	156 (44.5)	30 (8.5)	49 (20.5)	6 (2.5)

Data are given as number (percent) unless otherwise indicated.

a There was only one child in the 0–2 age group at the Bangladesh rural site, hence a standard deviation value could not be calculated.

SD, standard deviation.

### Discriminative Power of Survey Tools

Sensitivity, specificity, and 95% CIs for the various instruments that were studied are presented in Tables 2 and 3.

**Table 2:** Discriminative power of DHS/MICS questions about suspected pneumonia and of video for identifying childhood pneumonia (based on 2-wk and 4-wk recall).

Recall Period/Site	Diagnostic Validity	DHS Questions	MICS Questions	Video
**2-wk recall period**				
Pakistan (urban)	Sensitivity	64.7 (58.4–70.9)	63.8 (57.5–70.0)	59.8 (53.3–66.2)
	Specificity	68.5 (62.5–74.4)	67.2 (61.1–73.2)	78.0 (72.6–83.3)
Bangladesh (urban)	Sensitivity	24.6 (17.5–32.9)	25.4 (18.2–33.8)	26.9 (19.5–35.4)
	Specificity	81.7 (73.6–88.1)	82.5 (74.5–88.8)	82.5 (74.5–88.8)
Bangladesh (rural)	Sensitivity	71.1 (61.0–79.9)	70.1 (60.0–79.0)	26.8 (18.3–36.8)
	Specificity	56.5 (45.3–67.2)	56.5 (45.3–67.2)	77.6 (67.3–86.0)
**4-wk recall period**				
Pakistan (urban)	Sensitivity	71.4 (62.7–80.0)	69.5 (60.6–78.3)	64.8 (55.6–73.9)
	Specificity	69.4 (55.4–77.9)	67.6 (60.7–78.2)	74.8 (66.7–82.8)
Bangladesh (urban)	Sensitivity	23.2 (15.8–32.1)	23.2 (15.8–32.1)	28.6 (20.4–37.9)
	Specificity	82.7 (74.3–89.3)	83.6 (75.4–90.3)	80.9 (72.3–87.8)
Bangladesh (rural)	Sensitivity	72.3 (62.5–80.7)	73.3 (63.5–81.6)	29.7 (21.0–39.6)
	Specificity	53.2 (43.4–62.7)	53.2 (43.4–62.7)	83.8 (75.6–90.1)
**Overall**				
Pakistan (urban)	Sensitivity	66.9 (61.8–71.9)	65.7 (60.5–70.8)	61.4 (56.1–66.6)
	Specificity	68.8 (63.8–73.7)	67.3 (62.3–72.2)	77.0 (72.5–81.4)
Bangladesh (urban)	Sensitivity	24.0 (18.7–29.9)	24.4 (19.1–30.3)	27.7 (22.1–33.8)
	Specificity	82.2 (76.6–86.9)	83.0 (77.6–87.7)	81.7 (76.1–86.5)
Bangladesh (rural)	Sensitivity	71.7 (64.9–77.9)	71.7 (64.9–77.9)	28.3 (22.1–35.1)
	Specificity	54.6 (47.3–61.7)	54.6 (47.3–61.7)	81.1 (74.9–86.3)

**Table 3:** Sensitivity and specificity of different tools (individually and in combination).

Tools	Pakistan (Urban)		Bangladesh (Urban)		Bangladesh (Rural)	
	Sensitivity	Specificity	Sensitivity	Specificity	Sensitivity	Specificity
Fever^a^	83.0 (72.9–87.3)	31.8 (25.1–34.8)	80.6 (75.0–85.4)	26.5 (20.9–32.7)	86.4 (80.8–90.8)	23.0 (17.3–29.5)
LCI^a^	42.6 (37.2–47.9)	89.2 (85.9–92.4)	22.7 (17.6–28.5)	83.0 (77.6–87.7)	41.4 (34.5–48.6)	72.4 (65.6–78.6)
Chest pain^a^	46.5 (41.1–51.8)	77.8 (73.4–82.1)	0.4 (0.0–2.3)	97.0 (93.8–98.8)	3.0 (1.12–6.5)	97.4 (94.1–99.2)
DHS+fever	61.2 (56.1–66.6)	72.7 (68.2–77.6)	19.4 (14.6–25.0)	85.7 (80.4–89.9)	62.6 (55.5–69.4)	63.8 (56.6–70.5)
DHS+LCI	33.6 (28.6–38.8)	93.3 (90.6–95.9)	11.6 (7.8–16.3)	90.9 (86.4–94.3)	34.8 (28.2–41.9)	78.6 (72.2–84.1)
DHS+video	44.7 (39.3–50.0)	88.3 (84.9–91.7)	14.5 (10.3–19.5)	92.2 (87.9–95.3)	25.8 (19.8–85.2)	85.2 (79.4–89.9)
DHS+chest pain	39.5 (34.2–44.7)	86.6 (83.0–90.2)	0.4 (0.0–2.8)	99.6 (97.6–100.0)	3.0 (1.1–6.5)	98.5 (95.6–99.7)
DHS+fever+video	42.6 (36.8–48.3)	89.2 (85.9–92.4)	10.7 (7.1–15.3)	93.9 (90.0–96.6)	21.7 (16.2–28.1)	86.7 (81.2–91.1)
DHS+fever+LCI	24.9 (20.2–29.5)	95.6 (93.4–97.7)	9.1 (5.8–13.4)	93.5 (89.5–96.3)	30.8 (24.5–37.7)	84.7 (78.9–89.4)

Data are given as percent (95% CI). Results for both recall periods combined.

a These variables from the pneumonia score were selected for this analysis because they had the strongest association with pneumonia in screening univariate analyses.

LCI, lower chest indrawing.

Data are given as percent (95% CI).

#### DHS/MICS questions

Results at both sites showed poor discriminative power for DHS and MICS questions. Individual patterns of sensitivity and specificity varied substantially by study site and between urban and rural sites (Table 2). Results were similar for DHS and MICS questions, although not identical, because of some differences in questionnaire skip patterns. There was no significant difference in the validity of DHS and MICS questions at 2- versus 4-wk follow-up intervals (Table 2).

#### Pneumonia score

**Figure 4:**
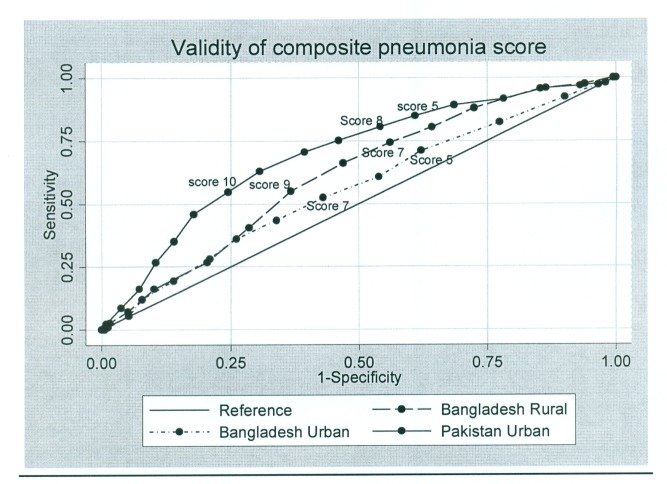
Validity of composite pneumonia score.

As expected, specificity increased and sensitivity declined with increasing pneumonia score, i.e., the number of positive symptoms from among the pneumonia score questions. An area-under-the-curve analysis was performed for the pneumonia score (see below) and is presented in Figure 4. Thresholds could be chosen so that the performance was comparable to DHS/MICS questions (e.g., >9 in Bangladesh) or to selectively increase test specificity.

#### Video tool

In Pakistan (where the video was developed and child images recorded), the video tool had a much higher discriminative power than in Bangladesh. There was no significant difference in recall with the video tool at 2 versus 4 wk in any of the three sites (Table 2).

### Recall of Antibiotic Use

In children with pneumonia, antibiotic treatment was recalled correctly by 66.0% and 66.8% of caregivers using DHS or MICS questions in Pakistan and Bangladesh, respectively, with no significant difference between 2- and 4-wk recall. Correct recall increased to 72.0% using a drug flip chart/photo album and to 71.3% using a computer-based drug chart in Pakistan, and to 78.8% and 81.2%, respectively, in Bangladesh.

### Association of Caregiver's Recall with Socio-Demographic Characteristics

In both Bangladesh study sites, but not in Pakistan, test specificities were higher for mothers who had higher levels of education. At the Bangladesh rural site the test specificity was 59% for mothers who had secondary or higher education, compared to a specificity of 37% for mothers whose education level was below primary. However, at the Bangladesh urban site these figures were 86% and 73%, respectively.

## Discussion

### Importance of Monitoring Antibiotic Treatment of Pneumonia

Despite recent falls in pneumonia mortality over the past ten years, it remains the largest single cause of death in children [8]. It is therefore essential that global and national programs track the coverage of effective interventions against pneumonia if Millennium Development Goal 4 is to be achieved. The antibiotic treatment of pneumonia is highly effective and, together with immunization, is one of the two main control strategies [9]. DHS and MICS household surveys are the primary tools used to measure intervention coverage in low- and middle-income countries where health information systems are weak, and are the primary source of information on common childhood illnesses and treatment coverage. We report here on the first study evaluating the validity of the estimates of pneumonia treatment coverage produced by the DHS and MICS survey instruments and discuss the implications of these findings for program monitoring.

### Validity of DHS/MICS Data for Estimation of Pneumonia Prevalence

Community-based epidemiological studies have estimated pneumonia incidence to be about 0.3 episodes per child per year in children under 5 y in low- and middle-income countries, which is some 12–18 times lower than the reported incidence of upper respiratory infections in this age group [10]. This low prevalence of pneumonia among all children with cough who are surveyed by DHS and MICS requires that the survey instruments must have very high specificity to identify pneumonia, otherwise the great majority of cases identified in the survey will not represent true cases of pneumonia (as reviewed by Campbell and colleagues in this *PLOS Medicine* Collection [11]). An example of this effect can be seen in a recent Pakistan DHS survey, which reported in 2006–2007 that 2,508/8,367 (29.9%) children under 5 y had cough and 1,178/8,367 (14.1%) had symptoms consistent with pneumonia [12]. This ratio of children with reported symptoms and signs of “cough and cold only” versus “suspected pneumonia” of 2∶1 is in marked contrast to that reported in community studies, where this ratio is typically very much higher, as noted above [13].

DHS and MICS reports clearly caution that these data (denoted as “suspected pneumonia” in MICS surveys) should not be used as a proxy measure of the prevalence of pneumonia in the community. However, many pneumonia control programs in the developing world, faced with a lack of data on this important parameter for planning purposes, use the information in this way. The results of our study show that the specificity of these survey tools is well below the very high levels required for this proxy to give an accurate estimation of pneumonia prevalence, and reinforce that these data should not be used for this purpose as they will lead to large overestimations of pneumonia prevalence [11].

### Validity of DHS/MICS Data for Estimation of Proportion of Children with Pneumonia Who Receive Antibiotic Treatment

DHS and MICS surveys identify children whose caregivers report that they had symptoms and signs consistent with pneumonia, and then ask whether these children were treated with an antibiotic. The validity of this proxy indicator of the proportion of children with pneumonia who receive antibiotic treatment is therefore entirely dependent on the validity of DHS/MICS-reported symptoms and signs of pneumonia as a measure of true pneumonia. For this to represent a valid denominator for this important indicator, it is important that a high proportion of children with “symptoms of acute respiratory infection” (DHS)/“suspected pneumonia” (MICS) actually have “true” pneumonia. If this proportion is low, then results of monitoring antibiotic coverage among these children will not only be inaccurate but also misleading. The results of this study show that DHS and MICS surveys at both sites have poor discriminative power for the identification of episodes of pneumonia in young children. Previous studies have shown that fever is a significant predictor of pneumonia and that adding fever to WHO criteria increased the specificity of pneumonia diagnosis [14],[15]. Our findings support this observation, as specificity increased when a question on the presence of fever was included (Table 3). Since DHS/MICS surveys currently collect information on the presence of fever in the last 2 wk, these data could be readily incorporated into the definition of “suspected pneumonia.” However, the addition of questions on the presence of fever made only modest improvements to the discriminative power. Thus, most children with “suspected pneumonia” (MICS) or “symptoms of acute respiratory infection” (DHS) who form the denominator for the “antibiotic treatment rate” do not truly have pneumonia. We conclude, therefore, that the addition of questions on the presence of these symptoms and signs would not make sufficiently large improvements in instrument validity for these to be adopted at present.

We found no significant difference in caregivers' recall at 2 and 4 wk, which is contrary to the findings in some previous studies. The recall sensitivity of caregivers' reports dropped when the interview took place after more than 2 wk in a respiratory questionnaire validation study conducted in Peru [6]. Some previous studies on maternal recall of breastfeeding duration showed a drop in mothers' recall accuracy with time [16],[17]. A more recent study conducted in Kenya also assessed the accuracy of caregivers' recall over time and found that it decreased when the recall period was increased to 2 wk from 3–4 d [18]. In our study, the lack of fall in performance with the longer recall period suggests that DHS/MICS surveys may be able to adopt a longer recall period for this question, thus increasing the period prevalence of true pneumonia cases detected in the survey. This possibility should be studied further, because since increasing the recall period, and thus the true period prevalence, can be expected to improve the validity of the indicator [11].

The sensitivities and specificities of the DHS/MICS survey instruments varied substantially by study site and by urban and rural setting (Table 2), reflecting different cultural factors, such as caregiver education, and making comparisons across countries or time periods difficult to interpret. These results have important implications for monitoring programs because these instruments are used to track progress over time (typically with serial surveys in different populations within a country) and to identify countries that have low intervention coverage in comparison to others. These findings urge caution in using DHS/MICS data for these purposes.

We show that the use of video material shows some potential to complement current DHS/MICS surveys and result in increased specificity for identification of episodes of pneumonia. However, substantial improvements in test specificity were found only in Pakistan, where the video was made, and were not found in Bangladesh. This finding is not unexpected and may relate to the fact that Pakistani children look somewhat different from Bangladeshi children, which is likely to have influenced caregiver responses. Moreover, we elected a priori to focus the video on showing fast breathing and lower chest indrawing. Ethnographic studies from this region have shown that mothers have varying perceptions and concepts regarding childhood pneumonia, and many caregivers do not associate fast breathing with pneumonia [19]–[21]. The fixed sequence of clips might also have influenced caregiver responses. The potential use of this video tool presents practical and logistical challenges, not least of which is that each individual country may be required to develop their own video. The improved discriminative power with the use of the video in Pakistan is promising, and we recommend that video presentation be explored further in an attempt to further improve its performance.

In children with pneumonia, antibiotic treatment was recalled correctly by two-thirds of caregivers using DHS or MICS questions in Pakistan and Bangladesh, with no significant difference between 2- and 4-wk recall. Correct recall increased with a drug flip chart/photo album or a computer-based drug chart. Thus, the validity of the DHS and MICS questions in correctly identifying antibiotic treatment for pneumonia improved with specific and more structured questions about the medicines or with the use of illustrations of common treatments. Although drug charts performed better than DHS/MICS questions at all three sites, using them in periodic household surveys may be challenging. There may be a very large number of available medicines in any given area, and new medicine brands are introduced often, so frequent revisions would be required.

### Study Limitations

The findings of the survey may have been biased by some aspects of the study design. DHS and MICS surveys are conducted in very diverse urban and rural settings. We attempted to reflect this through the urban and rural study sites selected for study. However, it is possible that the rural site in Bangladesh is not typical because of the ongoing research studies in that population. Furthermore, the urban site in Bangladesh included patients from an expanded urban and peri-urban catchment area, resulting in a relatively high rate of loss to follow-up and delayed follow-up. Thus, our findings may not be generalizable to some settings in low- and middle-income countries. Further studies, in particular in settings with high *Plasmodium falciparum* malaria transmission to explore the generalizability and to check the consistency of these findings, are recommended on this important issue. Nevertheless, the fact that our main study conclusions were confirmed in all three study settings suggests that the main findings may be widely relevant.

We selected study sites with investigators highly experienced in similar studies and employed a single technical expert to conduct the clinical training and oversee the monitoring of research staff in an attempt to reduce the potential for artifactual differences in study implementation across sites. Nevertheless, some of the differences shown between study sites may reflect some variation in application of methods across sites.

Mothers who take their children to a secondary or tertiary care center do not represent a random sample of all mothers who take part in DHS and MICS surveys. This is likely to influence results. We expect that these mothers will be more educated and show better recognition of signs of pneumonia in their children. To the extent that this is true, we may have overestimated the sensitivity and specificity of DHS/MICS questions. Furthermore, it is also likely that attendance at a hospital makes the episodes more memorable (and thus subject to better recall by the caregiver) and thus may once again lead to overestimation of specificity.

It is possible that the “case mix” in the hospital setting is different from that found in community surveys. We attempted to minimize this by excluding cases of severe pneumonia (rarely found in community surveys due to very low 2-wk period prevalence) and by adopting a matched group who had a respiratory illness (cough and cold) rather than studying healthy children. Nevertheless, this may have influenced our estimates of sensitivity and specificity. Thus, study procedures may have influenced caregiver recall. However, our main finding is that specificity levels associated with DHS and MICS questions are much too low to provide a robust basis for a pneumonia treatment rate indicator. We believe that the biases noted above tend to lead to an overestimation of specificity, and so we consider that our main conclusions about poor discriminative ability should remain valid. In addition, the level of specificity that we found is much lower than would be required, suggesting moderate effects of bias.

### Conclusions

Monitoring antibiotic treatment of pneumonia is essential for national and global programs. Despite this, we believe this is the first study to assess the validity of current surveys to measure this program indicator. Given the very large investment of human and financial resources in these surveys and their importance to child health programs, it is remarkable that there has been so little published data from research on this key issue. In the context of a low true prevalence of pneumonia, the DHS/MICS questions to define children with “suspected pneumonia” (MICS term) has a low yield for pneumonia, and thus most of these children do not have true pneumonia. This finding reinforces DHS and MICS recommendations that these measures not be used as a proxy for pneumonia prevalence, as using these measures will result in substantial overestimates.

We found the discriminative power (in particular the specificity) of a construct of caregiver-reported symptoms and signs of acute respiratory infection to be low for true pneumonia, as measured by DHS and MICS questionnaires; therefore, the use of such data from these surveys can be misleading for measuring antibiotic treatment rates. Using such data could lead to incorrect policy decisions having programmatic implications. Although the alternative tools evaluated in this study did not perform markedly better than DHS and MICS questions, we have presented some options for improving their specificity. Furthermore, the sensitivities and specificities of DHS and MICS survey instruments varied substantially by site, reflecting different cultural factors, such as caregiver education, and making comparisons across countries or time periods difficult to interpret. These results suggest that data from these surveys should not be used for the purpose of monitoring antibiotic treatment rates in children with pneumonia at the present time.

## Supporting Information

Text S1 **Pneumonia module of DHS questionnaire used in Pakistan.**
(DOC)

Text S2 **Pneumonia module of MICS questionnaire used in Pakistan.**
(DOC)

Text S3 **Bangla version of follow-up questionnaire used in Bangladesh.**
(PDF)

Text S4 **STARD checklist for reporting of studies of diagnostic accuracy.**
(DOC)

Text S5 **Original study protocol.** Validation of caregiver report of childhood pneumonia and antibiotic treatment from DHS and MICS data and assessment of alternative approaches.
(DOC)

## Abbreviations

DHS: Demographic and Health Survey/s
MICS: Multiple Indicator Cluster Survey/s
WHO: World Health Organization
