# Supplementary material for: Measuring Coverage in MNCH: A Prospective Validation Study in Pakistan and Bangladesh on Measuring Correct Treatment of Childhood Pneumonia
Source: PLoS Med. 2013 May 7;10(5):e1001422. doi: 10.1371/journal.pmed.1001422 (PMC3646205; doi:10.1371/journal.pmed.1001422)
Supplement: Text S1 — Pneumonia module of DHS questionnaire used in Pakistan. (DOC) [file pmed.1001422.s001.doc]

**DHS QUESTIONNAIRE**

1. *Has (NAME) been ill with a fever at any time in the last 2 weeks?*


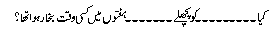


*Yes 1*

*No 2*

*Don’t Know 8*

1. *Has (NAME) had an illness with a cough at any time in the last 2 weeks?*

*
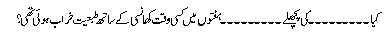
*

*Yes 1*

*No 2*

*Don’t Know 8*

1. *When (NAME) had an illness with a cough, did he/she breathe faster than usual with short, rapid breaths or have difficulty breathing?*

*
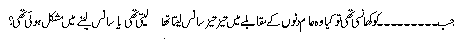
*

*Yes 1*

*No 2*

*Don’t Know 8*

1. *Was the fast or difficult breathing due to a problem in the chest or to a blocked or runny nose?*

*
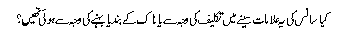
*

*Chest Only 1*

*Nose Only 2*

*Both 8*

*Other _______________ (Specify) 6*

*Don’t Know 8*

1. *Check 533: Had Fever?*


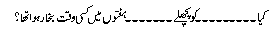


1. *Now I would like to know how much (NAME) was given to drink (including breastmilk) during the illness with a (fever/cough). Was he/she given less than usual to drink, about the same amount, more than usual to drink?*

*If less, probe: Was he/she given much less than usual to drink or somewhat less?*

*
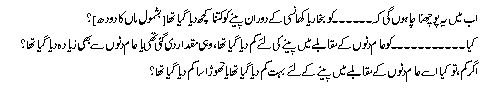
*

*Much Less 1*

*Somewhat Less 2*

*About the Same 3*

*More 4*

*Nothing to Drink 5*

*Don’t Know 6*

1. *When (NAME) had a (fever/cough), was he/she given less than usual to eat, about the same amount, more than usual, or nothing to eat?*

*If less, probe: Was he/she given much less than usual to eat or somewhat less?*

*
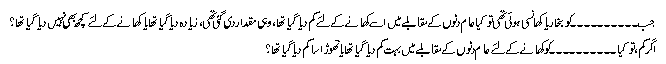
*

*Much Less 1*

*Somewhat Less 2*

*About the Same 3*

*More 4*

*Stopped Food 5*

*Never Gave Food 6*

*Don’t Know 6*

1. *Did you seek advice or treatment for the illness from any source?*

*
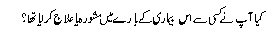
*

*Yes 1*

*No 2*

1. *Where did you seek advice or treatment? Anywhere else?*

*Probe to identify each type of source and circle the appropriate code(s).*

*If unable to determine if a hospital, health center or clinic is public or private medical, write the name of the place.*

*(Name of place)*

*
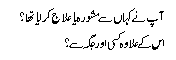
*

***Public Sector***

*Govt Hospital A*

*Govt Health Center B*

*Govt Health Post C*

*Mobile Clinic D*

*Fieldworker E*

*Other Public F*

*________________ (specify)*

***Private Medical Sector***

*PVT Hospital/Clinic G*

*Pharmacy H*

*PVT Doctor I*

*Mobile Clinic J*

*Fieldworker K*

*Other Private Med. L*

*________________ (specify)*

***Other Source***

*Shop M*

*Traditional Practitioner N*

*Other X*

*________________ (specify)*

1. *Where did you first seek treatment? Use letter code from 541.*

*
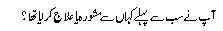
*

1. *How many days after the illness began did you first seek advice or treatment for (NAME)? If the same day, record ‘00’.*

*
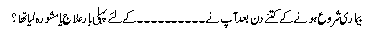
*

1. *Is (NAME) still sick with (fever/cough)?*

*
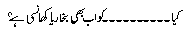
*

*Fever Only 1*

*Cough Only 2*

*Both Fever and Cough 3*

*No, Neither 4*

*Don’t Know 5*

1. *At any time during the illness, did (NAME) take any drugs for the illness?*

*
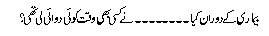
*

*Yes 1*

*No 2*

1. *What drugs did (NAME) take? Any other drugs? Record all mentioned.*

*
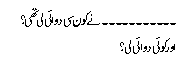
*

*Antimalarial Drugs*

*SP/Fansidar A*

*Chloroquine B*

*Amodiaquine C*

*Quinine D*

*Combination with Artemisinin E*

*Country Spec. CBD Antimalarial F*

*Other Antimalarial G*

*________________ (specify)*

*Antibiotic Drugs*

*Pill/Syrup H*

*Injection I*

*Other Drugs*

*Aspirin J*

*Acetaminophen K*

*Ibuprofen L*

*Other ________________ (specify) X*

*Don’t Know Z*

*Followed by specific questions about each drug mentioned…*
