# Supplementary material for: Measuring Coverage in MNCH: A Prospective Validation Study in Pakistan and Bangladesh on Measuring Correct Treatment of Childhood Pneumonia
Source: PLoS Med. 2013 May 7;10(5):e1001422. doi: 10.1371/journal.pmed.1001422 (PMC3646205; doi:10.1371/journal.pmed.1001422)
Supplement: Text S2 — Pneumonia module of MICS questionnaire used in Pakistan. (DOC) [file pmed.1001422.s002.doc]

**MICS QUESTIONNAIRE**

| CA7. At any time in the last two weeks, has (name) had an illness with a cough?  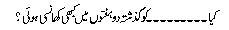 | Yes 1  No 2  DK 8 | 2⇨CA14  8⇨CA14 |
| --- | --- | --- |
| CA8. When (name) had an illness with a cough, did he/she breathe faster than usual with short, rapid breaths or have difficulty breathing?  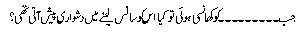 | Yes 1  No 2  DK 8 | 2⇨CA14  8⇨CA14 |
| CA9. Was the fast or difficult breathing due to a problem in the chest or a blocked or runny nose?  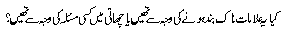 | Problem in chest 1  Blocked or runny nose 2  Both 3  Other (specify) 6  DK 8 | 2⇨CA14  6⇨CA14 |
| CA10. Did you seek any advice or treatment for the illness from any source?  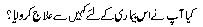 | Yes 1  No 2  DK 8 | 2⇨CA12  8⇨CA12 |
| CA11. FROM WHERE DID YOU SEEK ADVICE OR TREATMENT?  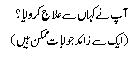 |  |  |
| CA12. Was (name) given any medicine to treat this illness?  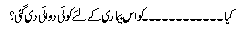 | Yes 1  No 2  DK 8 | 2⇨CA14  8⇨CA14 |
| CA13. What medicine was (name) given?  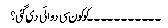  Probe:  Any other medicine?  Circle all medicines given. Write brand name(s) of all medicines mentioned.    (Names of medicines) | Antibiotic  Pill / Syrup A  Injection B  Anti-malarials M  Paracetamol / Panadol / Acetaminophen P  Aspirin Q  Ibuprofen R  Other (specify) X  DK Z |  |
