# Supplementary material for: Measuring Coverage in MNCH: A Prospective Validation Study in Pakistan and Bangladesh on Measuring Correct Treatment of Childhood Pneumonia
Source: PLoS Med. 2013 May 7;10(5):e1001422. doi: 10.1371/journal.pmed.1001422 (PMC3646205; doi:10.1371/journal.pmed.1001422)
Supplement: Text S3 — Bangla version of follow-up questionnaire used in Bangladesh. (PDF) [file pmed.1001422.s003.pdf]

HOME FOLLOW-UP QUESTIONNAIRE
Validation of caregiver report of childhood pneumonia and antibiotic treatment from Demographic and Health Survey (DHS) and Multiple Indicator Cluster Survey (MICS) data and assessment of alternative approaches

সেকসন A : শিশুর পরিচিতিমূলক তথ্যাবলী

সাইট আই. ডিঃ ☐☐ চিকিৎসকের আই.ডিঃ ☐☐ সিরিয়াল নং: ☐☐☐☐☐

A1. শিশুর (পূর্ণ) নাম... ....

A2. চলতি আই. ডি* (গ্রাম/বাড়ি/খানা/সিরিয়াল নং) ☐☐☐/☐☐☐/☐☐/☐☐
*শুধুমাত্র মির্জাপুর এর জন্য

A3. লিঙ্গ (ছেলে= 1 মেয়ে= 2) ☐

A4. জন্মতারিখ (দিন/মাস/বছর) ☐☐/☐☐/☐☐☐☐

A5. বয়স (মাস/ দিন) ☐ ☐/☐☐

A6. মাতার নাম ....

A7. পিতার নাম ....

A8. শিশুর সাথে সাক্ষাৎকার প্রদানকারীর সম্পর্ক ☐☐

A9. বর্তমান ঠিকানাঃ
বাড়িঃ ....
গ্রাম: ....
ইউনিয়ন: ....

A10. ভিজিট এর তারিখ এবং ফলাফল

	১৫ তম দিনে ভিজিট			২৯ তম দিনে ভিজিট		
	ভিজিট ১	ভিজিট ২	ভিজিট ৩	ভিজিট ১	ভিজিট ২	ভিজিট ৩
তারিখ	☐☐/☐☐ ☐☐☐☐	☐☐/☐☐ ☐☐☐☐	☐☐/☐☐ ☐☐☐☐	☐☐/☐☐ ☐☐☐☐	☐☐/☐☐ ☐☐☐☐	☐☐/☐☐ ☐☐☐☐
সময়	☐☐:☐☐	☐☐:☐☐	☐☐:☐☐	☐☐:☐☐	☐☐:☐☐	☐☐:☐☐
ফলাফল	☐☐	☐☐	☐☐	☐☐	☐☐	☐☐

মাঠ পর্যায়ে গবেষণা সহকারীর নাম .... আই.ডি: ☐☐

স্বাক্ষর ....

সেকসন B ঃ ডি. এইচ.এস প্রশ্নমালা

B1. (নাম) এর গত ....সপ্তাহের মধ্যে কি কখনও জ্বর হয়েছিল?

হ্যাঁ =1
না = 2
জানিনা =8

B2. (নাম) এর গত ....সপ্তাহের মধ্যে কি কখনও কাশির রোগ (ব্যারাম) হয়েছিল?

হ্যাঁ =1
না = 2 → B5 নং প্রশ্নে যান
জানিনা =8 → B5 নং প্রশ্নে যান

B3. (নাম) এর গত ....সপ্তাহের মধ্যে কাশিসহ অসুস্থতার সময় স্বাভাবিকের চেয়ে দ্রত, ঘন ঘন শ্বাস-প্রশ্বাস বা শ্বাস-কষ্ট হয়েছিল কি?

হ্যাঁ =1
না = 2 → B6 নং প্রশ্নে যান
জানিনা =8 → B6 নং প্রশ্নে যান

B4. (নাম) এর ঘন ঘন শ্বাস বা শ্বাস-কষ্ট কি বুকে সমস্যার কারনে নাকি নাক দিয়ে পানি ঝরা বা সর্দিতে নাক বন্ধ হবার কারনে হয়েছিল?

বুকের সমস্যা	1--
সর্দিতে নাক বন্ধ হওয়া	2--
উভয়ই	3--
অন্যান্য _________________ (নির্দিষ্ট করুন)	4--
জানিনা	8--

→ B6 নং প্রশ্নে যান

B5. যাচাই করুন(B1): জ্বর হয়েছিল কি না?

হ্যাঁ =1
না = 2
জানিনা =8

B6. অসুস্থতার (জ্বর বা কাশি) সময়ে (নাম)-কে কতটুকু তরল (বুকের দুধ সহ) পান করতে দেয়া হয়েছিল আমি সেই সম্পর্কে কিছু জানতে চাইব?
অসুস্থতার (জ্বর বা কাশি) পূর্বে তাকে যে পরিমাণ পানীয় পান করতে দেয়া হতো তাকে কি একই পরিমাণ পানীয় পান করতে দেয়া হয়েছিল , নাকি তার চেয়ে কম বা বেশি পানীয় পান করতে দেয়া হয়েছিল?

স্বাভাবিকের চেয়ে কম হলে প্রোব (Probe) করুন: তাকে কি স্বাভাবিকের চেয়ে খুবই কম পানীয় পান করতে দেয়া হয়েছিল নাকি কিছুটা কম পানীয় পান করতে দেয়া হয়েছিল?

খুবই কম	1
কিছুটা কম	2
একই পরিমাণ	3
বেশি	4

কিছুই পান করতে দেয়া হয় নাই	5
জানি না	8

B7. অসুস্থতার (জ্বর বা কাশি) সময়ে (নাম)- কে কতটুকু শক্ত খাবার খেতে দেয়া হয়েছিল? স্বাভাবিকের চেয়ে কম, প্রায় স্বাভাবিক নাকি স্বাভাবিকের চেয়ে বেশি শক্ত খাবার খেতে দেয়া দেয়া হয়েছিল?

স্বাভাবিকের চেয়ে কম হলে প্রোব (Probe) করুন: তাকে কি স্বাভাবিকের চেয়ে অনেক কম শক্ত খাবার খেতে দেয়া হয়েছিল নাকি কিছুটা কম?

খুবই কম	1
কিছুটা কম	2
একই পরিমাণ	3
বেশি	4
খাবার খেতে দেয়া হয়নি	5
আধা শক্ত বা শক্ত খাবার খেতে দেয়া শুরু করিনি	6
জানি না	8

B8. (নাম)-এর এই অসুস্থতার জন্য পরামর্শ বা চিকিৎসা নিতে কারও কাছে গিয়েছিলেন কি?

হ্যাঁ	1
না	2→B12 নং প্রশ্নে যান

B9. আপনি পরামর্শ বা চিকিৎসা নিতে কোথায় গিয়েছিলেন/ দেখিয়েছিলেন?
প্রথমে কোথায়, তার পরে কোথায়?
একই স্থানে একাধিকবার বা একাধিক স্থান থেকে সেবা নিলে, সেবা নেয়ার ক্রমানুসারে বক্সে সেবা প্রতিষ্ঠানের কোড লিখুন।

যদি হাসপাতাল, স্বাস্থ্য কেন্দ্র, ক্লিনিক সরকারী না প্রাইভেট এটা সঠিকভাবে নির্ণয় করতে না পারে তবে স্থানের নাম লিখে রাখুন।

____

(স্থানের নাম)

	1	2	3	4
SEQUENCE OF CARE:				

(FILL UP THE BOXES ACCORDING TO THE SEQUENCE OF CARE RECEIVED).

বাড়ি

বাড়ি ....A

সরকারী প্রতিষ্ঠান

সরকারী হাসপাতাল ....B

বিশেষায়িত সরকারী হাসপাতাল __________________ C
(নির্দিষ্ট করুন)

জেলা হাসপাতাল ....D

মাতৃসদন...E

উপজেলা স্বাস্থ্যকেন্দ্র (UHC) ....F
স্বাস্থ্য ও পরিবার কল্যাণ কেন্দ্র (H&FWC)..............................G
স্যাটেলাইট ক্লিনিক /EPI Outreach সাইট.............................H
কমিউনিটি ক্লিনিক..I
সরকারী মাঠকর্মী (FWA)..J
অন্যান্য সরকারী প্রতিষ্ঠান ____________________...........K
(নির্দিষ্ট করুন)

এনজিও সেক্টর

এনজিও স্থায়ী কেন্দ্র L
এনজিও অস্থায়ী কেন্দ্র...M
এনজিও ডিপো হোল্ডার.. N
এনজিও মাঠকর্মী .... O
অন্যান্য এনজিও সেক্টর ___________________ .... P
(নির্দিষ্ট করুন)

প্রাইভেট মেডিকেল প্রতিষ্ঠান

প্রাইভেট হাসপাতাল/ ক্লিনিক...Q
পাশ করা ডাক্তার..R
সনাতন চিকিৎসকের চেম্বার..S
ফার্মেসি / ঔষধের দোকান...T
প্রাইভেট মেডিকেল কলেজ হাসপাতাল_________________........U
(নির্দিষ্ট করুন)

অন্যান্য প্রাইভেট সেক্টর ____________________ V
(নির্দিষ্ট করুন)

অন্যান্য _____________________........ X
(নির্দিষ্ট করুন)

B10. (নাম)-এর এই অসুস্থতার কত দিন পর প্রথম পরামর্শ বা চিকিৎসা নিতে কারও কাছে গিয়েছিলেন? একই দিন হলে ”০০” লিখুন।

☐☐ দিন

B11. (নাম) কি এখনও অসুস্থ (জ্বর বা কাশি)?

শুধু জ্বর	1
শুধু কাশি	2
জ্বর বা কাশি উভয়ই	3
কোনটাই না	4
জানি না	8

B12. এই অসুস্থতার সময়ে (নাম) কে এই অসুস্থতার জন্য কোন ঔষধ খেতে দিয়েছিলেন?

হ্যাঁ =1
না = 2 → MICS (section C) প্রশ্নে যান
জানিনা =8 → MICS (section C) প্রশ্নে যান

B13. অসুস্থতার সময় (নাম) কি কি ঔষধ সেবন করেছে?

এছাড়া অন্য কোন ঔষধ?

প্রতিটি রেকর্ড করুন

ম্যালেরিয়ার প্রতিষেধক ঔষধ

এস পি/ ফ্যানসিডার... A
ক্লোরোকুইন .... B
এমোডায়াকুইন C
কুইনাইন .... D
আর্টেমেসিন.. E
কান্ট্রি স্পেসিফিক CBD অ্যান্টিম্যালেরিয়াল .... F
অন্যান্য ম্যালেরিয়ার প্রতিষেধক ___________________ G
(নির্দিষ্ট করুন)

(

এন্টিবায়োটিক ঔষধ

বড়ি/সিরাপ ....H
ইঞ্জেকশন I

অন্যান্য ঔষধ

এসপিরিন.. ....J
এসিটামিনোফেন ....K
আইবিউপ্রফেন L
অন্যান্য _____________________..................X
(নির্দিষ্ট করুন)

জানি না ....Z

প্রতিটি ঔষধ সম্পর্কে নিচের তথ্যগুলো জেনে নিন

১ নংঔষধ:

নাম...অথবা জানিনা
ঔষধের প্রস্তুতির ধরণ................... অথবা জানিনা
ঔষধের মাত্রা .... অথবা জানিনা
ঔষধের মেয়াদ....................... ...অথবা জানিনা

(নাম) কি ঔষধ সেবন করেছে?

হ্যাঁ,পুরোপুরি সেবন করেছে........1
না,আংশিক সেবন করেছে..........2
না, সেবন করে নাই.......3
এখনও চলছে.............4
জানি না..............8

২ নংঔষধ:

নাম...অথবা জানিনা
ঔষধের প্রস্তুতির ধরণ................... অথবা জানিনা
ঔষধের মাত্রা .... অথবা জানিনা
ঔষধের মেয়াদ............................ অথবা জানিনা

(নাম) কি ঔষধ সেবন করেছে?

হ্যাঁ,পুরোপুরি সেবন করেছে........1
না,আংশিক সেবন করেছে..........2
না, সেবন করে নাই.......3
এখনও চলছে.............4
জানি না..............8

৩ নংঔষধ:

নাম...অথবা জানিনা
ঔষধের প্রস্তুতির ধরণ................... অথবা জানিনা
ঔষধের মাত্রা .... অথবা জানিনা
ঔষধের মেয়াদ....................... ...অথবা জানিনা

৪ নংঔষধ:

নাম...অথবা জানিনা
ঔষধের প্রস্তুতির ধরণ................... অথবা জানিনা
ঔষধের মাত্রা .... অথবা জানিনা
ঔষধের মেয়াদ....................... ...অথবা জানিনা

	(নাম) কি ঔষধ সেবন করেছে?<br><br>হ্যাঁ,পুরোপুরি সেবন করেছে........1<br>না,আংশিক সেবন করেছে..........2<br>না, সেবন করে নাই.......3<br>এখনও চলছে.............4<br>জানি না...............8	(নাম) কি ঔষধ সেবন করেছে?<br><br>হ্যাঁ,পুরোপুরি সেবন করেছে........1<br>না,আংশিক সেবন করেছে..........2<br>না, সেবন করে নাই.......3<br>এখনও চলছে.............4<br>জানি না...............8
B 14	কেউ কি এই ঔষধ খেতে পরামর্শ দিয়েছিল?	হ্যাঁ ....1<br>না 2
B 15	এই ঔষধ খেতে কে পরামর্শ দিয়েছিল বা প্রেসক্রাইব করেছিল?	**স্বাস্থ্য পেশাজীবী**<br>পাশ করা ডাক্তার ....A<br>নার্স/ধাত্রী/প্যারামেডিক ....B<br>পরিবার কল্যাণ পরিদর্শিকা (FWV)........C<br>কমিউনিটি স্কিলড বার্থ অ্যাটেনডেন্ট (CSBA) ....D<br>MA/ SACMO.................................E<br>স্বাস্থ্য সহকারী (HA)...........................F<br>পরিবার কল্যাণ সহকারী (FWA)............G<br><br>**অন্যান্য পেশাজীবী**<br>প্রশিক্ষণ প্রাপ্ত দাই ....H<br>সনাতন দাই ....I<br>পাশ না করা ডাক্তার ....J<br>ঔষধ বিক্রেতা.....................................K<br><br>**অন্যান্য**<br>ব্র্যাক কমিউনিটি কর্মী ....L<br>সূর্যের হাসি কর্মী ....M<br>অন্যান্য এনজিও মাঠকমী ....N<br><br>অন্যান্য ________________________X<br>(নির্দিষ্ট করুন)
B 16	আপনি কোথা থেকে এই ঔষধ নিয়েছিলেন?	**সরকারী প্রতিষ্ঠান**<br>সরকারী হাসপাতাল ....A<br>বিশেষায়িত সরকারী হাসপাতাল ....B<br>(নির্দিষ্ট করুন)<br>জেলা হাসপাতাল .... C<br>মাতৃসদন ....D

উপজেলা স্বাস্থ্যকেন্দ্র
(UHC)...E
স্বাস্থ্য ও পরিবার কল্যাণ কেন্দ্র (H&FWC).... F
স্যাটেলাইট ক্লিনিক /EPI Outreach সাইট
.... G
কমিউনিটি ক্লিনিক.................................. H
সরকারী মাঠকর্মী (FWA) I
অন্যান্য সরকারী প্রতিষ্ঠান
______________________ J

(নির্দিষ্ট করুন))

এনজিও সেক্টর

এনজিও স্থায়ী কেন্দ্র .... K
এনজিও অস্থায়ী কেন্দ্র L
এনজিও ডিপো হোল্ডার............................. M
এনজিও মাঠকর্মী .... N
অন্যান্য এনজিও সেক্টর _____________ O
(নির্দিষ্ট করুন)

প্রাইভেট মেডিকেল প্রতিষ্ঠান

প্রাইভেট হাসপাতাল/ ক্লিনিক ....P
পাশ করা ডাক্তার ....Q
সনাতন চিকিৎসকের চেম্বার ....R
ফার্মেসি / ঔষধের দোকান......................... S
প্রাইভেট মেডিকেল কলেজ
হাসপাতাল______________________ T

(নির্দিষ্ট করুন)

অন্যান্য প্রাইভেট সেক্টর

______________________ U
(নির্দিষ্ট করুন)

অন্যান্য উৎস:

দোকান V
বন্ধুবান্ধব/প্রতিবেশী..W
অন্যান্য X
(নির্দিষ্ট করুন)

সেকসন C : এম. আই. সি.এস . প্রশ্নমালা

C1.	গত ....সপ্তাহের মধ্যে (নাম) কোন সময় কাশি জনিত কারণে অসুস্থ হয়েছিল কি ?	হ্যাঁ .... 1<br>না .... 2<br>জানিনা... 8	
C2.	গত ....সপ্তাহে কাশিসহ অসুস্থতার সময় তার (নাম) স্বাভাবিকের চেয়ে দ্রুত শ্বাস প্রশ্বাস, ঘনঘন শ্বাস প্রশ্বাস অথবা শ্বাস কষ্ট হয়েছিল কি ?	হ্যাঁ .... 1<br>না .... 2<br>জানিনা 8	 → C4<br>→ C4
C3.	তার(নাম) এ দ্রুত শ্বাস প্রশ্বাস অথবা শ্বাস কষ্ট কি বুকের সমস্যা, নাকি বন্ধ নাক বা নাকের সর্দির কারণে হয়েছিল ?	বুকের সমস্যা .... 1<br>সর্দিতে নাক বন্ধ হওয়া .... 2<br>উভয়ই .... 3<br>অন্যান্য (উল্লেখ করুন) .... 6<br>জানিনা 8	
C4.	(নাম) এ অসুস্থতার জন্য আপনি ঘরের বাইরের কারও পরামর্শ বা চিকিৎসা সেবা নিয়েছিলেন কি ?	হ্যাঁ .... 1<br>না.. 2<br>জানিনা 8	
C5.	কোথা থেকে এ সেবা নিয়েছেন ?<br>*(একাধিক উত্তর হতে পারে) ।কিন্তু মা কে কোন সূত্র দেবেন না।*<br><br>যদি হাসপাতাল, স্বাস্থ্যকেন্দ্র অথবা ক্লিনিক থেকে সেবা নিয়ে থাকেন, তাহলে নিম্নোক্ত স্থানে নাম লিখুন।যথাযথ স্থানটি খুঁজে বের করার জন্য মাকে প্রোব করুন।<br><br>-----------------------------<br>(স্থানের নাম)	**সরকারী প্রতিষ্ঠান ঃ**<br>সরকারী হাসপাতাল/স্বাস্থ্য কেন্দ্র .... A<br>স্বাস্থ্য কর্মী/স্বাস্থ্য সহকারী .... D<br>ভ্রাম্যমাণ/প্রত্যন্ত ক্লিনিক .... E<br>অন্যান্য সরকারী (উল্লেখ করুন)................. H<br><br>**প্রাইভেট মেডিক্যাল প্রতিষ্ঠান ঃ**<br>প্রাইভেট হাসপাতাল/ক্লিনিক .... I<br>প্রাইভেট চিকিৎসক .... J<br>প্রাইভেট ফার্মেসী .... K<br>অন্যান্য প্রাইভেট চিকিৎসা<br>(উল্লেখ করুন)...................................... O<br><br>**অন্যান্য উৎসঃ**<br>আত্মীয় বা বন্ধু-বান্ধব .... P<br>দোকান... Q<br>পল্লী চিকিৎসক .... R<br>এনজিও হাসপাতাল/ক্লিনিক .... S<br>**অন্যান্য (উল্লেখ করুন)** X	
C6.	(নাম) কে তার এ অসুস্থতার চিকিৎসার জন্য ঔষধ দেয়া হয়েছিল কি ?	হ্যাঁ .... 1<br>না.. 2<br>জানি না 8	

C7. তাকে (নাম) কি কি ঔষধ দেয়া হয়েছিল ?

(একাধিক উত্তর হতে পারে)।

এন্টিবায়োটিক	
পিল/ সিরাপ......................	A
ইঞ্জেকশন	B
এন্টিম্যালেরিয়াল	M
প্যারাসিটামল/প্যানাডোল	P
এ্যাসপিরিন	Q
আইবুপ্রোফেন (Ibuprofen)	R
অন্যান্য (উল্লেখ করুন)	X
জানিনা	Z

সেকসন D : নিউমোনিয়া স্কোর প্রশ্নমালা

D1	(নাম) এর কি গত (-------------) সপ্তাহে কাশির অসুখ/ব্যারাম হয়েছিল?	হ্যাঁ / না/ জানিনা
D2	(নাম) এর কি গত (-------------) সপ্তাহে জ্বর হয়েছিল?	হ্যাঁ / না/ জানিনা
D3	(নাম) এর কি গত (---------) সপ্তাহে কাঁপুনি বা ঘাম হয়েছিল?	হ্যাঁ / না/ জানিনা
D4	(নাম) কি গত (--------) সপ্তাহে অস্থির ছিল?	হ্যাঁ / না/ জানিনা
D5	(নাম) কি গত (--------) সপ্তাহে খিটখিটে ছিল?	হ্যাঁ / না/ জানিনা
D6	(নাম) এর কি গত (--------) সপ্তাহে ক্ষুধামন্দা হয়েছিল?	হ্যাঁ / না/ জানিনা
D7	(নাম) এর কি গত (--------) সপ্তাহে ঘুম ঘুম ভাব ছিল বা ঘুম ভাঙ্গাতে কষ্ট হয়েছিল?	হ্যাঁ / না/ জানিনা
D8	(নাম) এর কি গত (--------) সপ্তাহে শ্বাসের সময় শোঁ শোঁ শব্দ শুনতে পেয়েছেন?	হ্যাঁ / না/ জানিনা
D9	গত (--------) সপ্তাহে (নাম) কি ঘন ঘন শ্বাস নিয়েছিল?	হ্যাঁ / না/ জানিনা
D10	গত (--------) সপ্তাহে (নাম) কি কখনও স্বাভাবিকের চেয়ে দ্রুত শ্বাস-প্রশ্বাস নিয়েছে?	হ্যাঁ / না/ জানিনা
D11	গত (--------) সপ্তাহে (নাম) এর নাকের পাটা ফুলে যেতে দেখেছেন?	হ্যাঁ / না/ জানিনা
D12	গত (--------) সপ্তাহে (নাম) এর পানির প্রতি অনীহা দেখেছেন বা স্বাভাবিকের চেয়ে কম পানি পান করতে দেখেছেন?	হ্যাঁ / না/ জানিনা
D13	গত (--------) সপ্তাহে (নাম) এর শ্বাসের সময় বুকের খাঁচা দেবে যেতে বা তলপেট ভিতরের দিকে ঢুকে যেতে দেখেছেন?	হ্যাঁ / না/ জানিনা
D14	গত (--------) সপ্তাহে (নাম) এর কি কখনও বুকে ব্যথা হয়েছিল?	হ্যাঁ / না/ জানিনা
D15	গত (--------) সপ্তাহে (নাম) এর কি কখনও শ্বাস নিতে কষ্ট হয়েছিল?	হ্যাঁ / না/ জানিনা
D16	গত (--------) সপ্তাহে (নাম) কি কখনও বমি করেছিল?	হ্যাঁ / না/ জানিনা
D17	গত (--------) সপ্তাহে (নাম) কি কখনও ঘড়ঘড় শব্দ করেছিল?	হ্যাঁ / না/ জানিনা
D18	গত (--------) সপ্তাহে (নাম) এর শরীরের চামড়ায় কোথাও কি নীল বর্ণ দেখেছিলেন (প্রধানত ঠোঁট এবং জিহ্বায়)?	হ্যাঁ / না/ জানিনা
D19	গত (--------) সপ্তাহে (নাম) এর কি কখনও কাশির সাথে রক্ত বের হয়েছিল?	হ্যাঁ / না/ জানিনা
D20	গত (--------) সপ্তাহে (নাম) কি কখনও অজ্ঞান হয়ে গিয়েছিল?	হ্যাঁ / না/ জানিনা

সেকসন E : ভিডিও অনুশীলনী

সাক্ষাৎকার গ্রহণকারী মা কে বলবেন
এখন আমি আপনাকে কম্পিউটারের মাধ্যমে একটি ভিডিও দেখাবো যেখানে ৩ টি শিশুর ছবি আপনি দেখতে পাবেন, যারা কাশি বা শ্বাসকষ্টে ভুগছে। এই শিশুগুলোকে আমরা ১, ২ ও ৩ নং দিয়ে চিহ্নিত করেছি। আপনি মনোযোগ দিয়ে পুরো ভিডিওটি দেখুন এবং মনে করার চেষ্টা করুন কোন ভিডিওটি(১ অথবা ২ অথবা ৩ নং) আপনার শিশুর (------------) সপ্তাহ আগের অসুস্থতাকালীন সময়ের লক্ষণগুলোর মত।

এরপর সাক্ষাৎকার গ্রহণকারী ভিডিও টি কম্পিউটারে দেখাবেন। ভিডিও দেখানোর সময় মা বা সাক্ষাৎকার গ্রহণকারী কেউই কোন বিঘ্ন ঘটাবেন না।

ভিডিও দেখানোর পরে সাক্ষাৎকার গ্রহণকারী নিচের প্রশ্নগুলো জিজ্ঞাসা করবেন

E1. কোন ভিডিওটি আপনার শিশুর (-----------) সপ্তাহ আগের অসুস্থতাকালীন সময়ের লক্ষণগুলোর মত।

প্রশ্ন করার সময় সাক্ষাৎকার গ্রহণকারী নিচের অনুশীলনী বক্সের উপযুক্ত স্থানে (√) চিহ্ন দিবেন।

ভিডিও অনুশীলনী

(সঠিক উত্তরটি খুঁজে বের করুন)

**সবুজ**<br>**(০- ২ মাস)**	**নীল**<br>**(২-১২ মাস)**	**লাল**<br>**(১২-৫৯ মাস)**
**১**	**১**	**১**
**২**	**২**	**২**
**৩**	**৩**	**৩**

E2. উপরের কোন নাম্বার কি অর্ন্তভূক্ত হয়েছে?
হ্যাঁ 1
না... 2

E3. মা কি ভিডিওটি পুনরায় দেখানোর জন্য অনুরোধ করেছেন?
হ্যাঁ 1
না... 2

সেকসন F : ড্রাগ চার্ট

সাক্ষাৎকার গ্রহণকারী মাকে বলবেন

(কম্পিউটার ভিত্তিক ড্রাগ চার্ট) এর জন্য

প্রথমে আমি আপনাকে কিছু ঔষধের ছবি কম্পিউটারে দেখাবো যা দেখে আপনার শিশুকে অসুস্থতাকালীন সময়ে যেসব ঔষধ খাওয়ানো হয়েছে তা যেন আপনি সনাক্ত করতে পারেন।

F1.	ছবিগুলি কম্পিউটারে মনোযোগ দিয়ে দেখুন এবং আপনার শিশুকে যেসব ঔষধ খাওয়ানো হয়েছে তা সনাক্ত করতে চেষ্টা করুন।(একাধিক উত্তর হতে পারে)

সাক্ষাৎকার গ্রহণের সময় সাক্ষাৎকার গ্রহণকারী ছবির নাম্বার দেখে মা এর উত্তর অনুযায়ী নাম্বারটি বৃত্তায়িত করবেন।

যে ঔষধ দেয়া হয়েছে (কম্পিউটার ভিত্তিক ড্রাগ চার্ট)

১	১৩	২৫	৩৭	৪৯	৬১
২	১৪	২৬	৩৮	৫০	৬২
৩	১৫	২৭	৩৯	৫১	
৪	১৬	২৮	৪০	৫২	
৫	১৭	২৯	৪১	৫৩	
৬	১৮	৩০	৪২	৫৪	
৭	১৯	৩১	৪৩	৫৫	
৮	২০	৩২	৪৪	৫৬	
৯	২১	৩৩	৪৫	৫৭	
১০	২২	৩৪	৪৬	৫৮	
১১	২৩	৩৫	৪৭	৫৯	
১২	২৪	৩৬	৪৮	৬০	

**F2. উপরের কোন নাম্বার কি অর্ন্তভূক্ত হয়েছে?**	হ্যাঁ .... 1<br>না... 2
**যদি হ্যাঁ হয়, তাহলে অর্ন্তভূক্ত নাম্বারের ঔষধ গুলোর ছবি আবার দেখান এবং প্রতিটি ঔষধের জন্য এই প্রশ্নগুলো করুন**	ঔষধের নং. _____: প্রতিবারে মাত্রা......................<br>দিনে কতবারঃ ....<br>কতদিনের জন্যঃ ,,,,,,,,,,,,,,,,,,,<br>ঔষধের নং. _____: প্রতিবারে মাত্রা......................<br>দিনে কতবারঃ ....<br>কতদিনের জন্যঃ ,,,,,,,,,,,,,,,,,,,<br>ঔষধের নং. _____: প্রতিবারে মাত্রা......................<br>দিনে কতবারঃ ....<br>কতদিনের জন্যঃ ,,,,,,,,,,,,,,,,,,,<br>ঔষধের নং. _____: প্রতিবারে মাত্রা......................<br>দিনে কতবারঃ ....<br>কতদিনের জন্যঃ ,,,,,,,,,,,,,,,,,,,

(অ্যালবাম ভিত্তিক ড্রাগ চার্ট) এর জন্য

সাক্ষাৎকার গ্রহণকারী মাকে বলবেন:

এখন আমি আপনাকে কিছু ঔষধের ছবি অ্যালবামে দেখাবো যা দেখে আপনার শিশুকে অসুস্থতাকালীন সময়ে যেসব ঔষধ খাওয়ানো হয়েছে তা যেন আপনি সনাক্ত করতে পারেন।

F3.	ছবিগুলি অ্যালবামে মনোযোগ দিয়ে দেখুন এবং আপনার শিশুকে যেসব ঔষধ খাওয়ানো হয়েছে তা সনাক্ত করতে চেষ্টা করুন।(একাধিক উত্তর হতে পারে)

সাক্ষাৎকার গ্রহণের সময় সাক্ষাৎকার গ্রহণকারী ছবির নাম্বার দেখে মা এর উত্তর অনুযায়ী নাম্বারটি বৃত্তায়িত করবেন।

যে ঔষধ দেয়া হয়েছে (অ্যালবাম ভিত্তিক ড্রাগ চার্ট)

১	১৩	২৫	৩৭	৪৯	৬১
২	১৪	২৬	৩৮	৫০	৬২
৩	১৫	২৭	৩৯	৫১	
৪	১৬	২৮	৪০	৫২	
৫	১৭	২৯	৪১	৫৩	
৬	১৮	৩০	৪২	৫৪	
৭	১৯	৩১	৪৩	৫৫	
৮	২০	৩২	৪৪	৫৬	
৯	২১	৩৩	৪৫	৫৭	
১০	২২	৩৪	৪৬	৫৮	
১১	২৩	৩৫	৪৭	৫৯	
১২	২৪	৩৬	৪৮	৬০	

F4. উপরের কোন নাম্বার কি অর্ন্তভূক্ত হয়েছে?

হ্যাঁ 1
না 2

যদি হ্যাঁ হয়, তাহলে অর্ন্তভূক্ত নাম্বারের ঔষধ গুলোর ছবি আবার দেখান এবং প্রতিটি ঔষধের জন্য এই প্রশ্নগুলো করুন

ঔষধের নং. _____: প্রতিবারে মাত্রা.....................
দিনে কতবারঃ ....
কতদিনের জন্যঃ ,,,,,,,,,,,,,,,,,,

ঔষধের নং. _____: প্রতিবারে মাত্রা.....................
দিনে কতবারঃ ....
কতদিনের জন্যঃ ,,,,,,,,,,,,,,,,,,

ঔষধের নং. _____: প্রতিবারে মাত্রা.....................
দিনে কতবারঃ ....
কতদিনের জন্যঃ ,,,,,,,,,,,,,,,,,,

ঔষধের নং. _____: প্রতিবারে মাত্রা.....................
দিনে কতবারঃ ....
কতদিনের জন্যঃ ,,,,,,,,,,,,,,,,,,

F5.আমরা আপনাকে কম্পিউটার ভিত্তিক এবং অ্যালবাম ভিত্তিক, দুই ধরণের ড্রাগ চার্ট দেখালাম। এখন আপনি আমাদের বলুন কোনটি আপনার কাছে বেশী উপযোগী বলে মনে হয়েছে।

কম্পিউটার ভিত্তিক ড্রাগ চার্ট 1
অ্যালবাম ভিত্তিক ড্রাগ চার্ট 2
দুটোই 3
কোনটিই নয় 4
অন্যান্য 5
(উল্লেখ করুন)

F6. যদি যেকোন একটি(কম্পিউটার ভিত্তিক ড্রাগ চাট/ অ্যালবাম ভিত্তিক ড্রাগ চার্ট) কে আপনি পছন্দ করে থাকেন তাহলে তার কারণ উল্লেখ করুন।

1. ………………………………………………
2. ……………………………………………….
3. ……………………………………………….
4. ………………………………………………..
5. …………………………………………………
