# Supplementary material for: Measuring Coverage in MNCH: A Prospective Validation Study in Pakistan and Bangladesh on Measuring Correct Treatment of Childhood Pneumonia
Source: PLoS Med. 2013 May 7;10(5):e1001422. doi: 10.1371/journal.pmed.1001422 (PMC3646205; doi:10.1371/journal.pmed.1001422)
Supplement: Text S5 — Original study protocol. Validation of caregiver report of childhood pneumonia and antibiotic treatment from DHS and MICS data and assessment of alternative approaches. (DOC) [file pmed.1001422.s005.doc]

**Validation of caregiver report of childhood pneumonia and antibiotic treatment from Demographic and Health Survey (DHS) and Multiple Indicator Cluster Survey (MICS) data and assessment of alternative approaches**

**General Introduction**

**Childhood pneumonia**

*Mortality*

Childhood pneumonia is the leading single cause of mortality in children age less than 5 years. It is responsible for about 17% of all deaths in children age less than 5 years, of which more than 70% take place in sub-Saharan Africa and South-east Asia. [Black 2010]

*Incidence of clinical pneumonia*

The estimated incidence of pneumonia in young children for developing countries was 0.28 episodes per child-year (interquartile range from 0.21–0.71) based on an analysis of data from selected 28 community-based longitudinal studies). [Rudan 2004]. Estimates of clinical pneumonia incidence are highest in South-East Asia (0.36 episodes per child year), followed by Africa (0.33 episodes per child year) and by the Eastern Mediterranean (0.28 episodes per child year) and lowest in the Western Pacific (0.22 episodes per child year), Americas (0.10 episodes per child year) and Europe (0.06 episodes per child year) [Rudan 2008]. Of all community-based episodes of pneumonia 7–13% are severe enough to be life-threatening and require hospitalization.

*Global Action Plan for the Prevention and Control of Pneumonia (GAPP)*

In response to the Millennium Development Goal 4 (MDG4) challenge of reducing the under-five mortality rate by two-thirds between 1990 and 2015, WHO/UNICEF have developed the Global Action Plan for the prevention and control of Pneumonia (GAPP). GAPP recommends that children be provided with healthy environments and access to preventive interventions to protect them from getting pneumonia and also to access to treatment if they do develop pneumonia. The specific aims of the GAPP are to:

- Reduce mortality due to pneumonia, in children under the age of 5, by 65% by 2015, compared with levels in 2000
- Reduce the incidence of severe pneumonia by 25% in children under the age of 5 by 2015, compared with levels in 2000

To achieve these goals it is necessary that levels of coverage of effective interventions against pneumonia increase rapidly. In order to monitor progress in coverage there is a need for valid measures of coverage. Although UNICEF, USAID and other agencies have invested heavily in efforts to track coverage through the development and implementation of the DHS and Multiple Indicator Cluster Surveys (MICS), as well as working to establish interagency consensus on the most important indicators to track over time, methodological weaknesses in current efforts to measure coverage (and especially coverage trends over time) have been highlighted. Some of these weaknesses are being addressed (e.g. the Institute for Health Metrics and Evaluation reports that they are working on a subset of 30 coverage indicators to improve assessments of “effective coverage”). However, there remains an urgent need to improve the validity and reliability of coverage measurement, across countries and over time.

Of particular importance are coverage indicators of *correct treatment of childhood infectious diseases*, especially those for pneumonia. Prompt treatment with a full course of antibiotics can reduce deaths from pneumonia by over 40%, and strategies to achieve high, sustained and equitable coverage for this intervention are receiving increasing attention. Despite its importance, efforts to measure antibiotic treatment coverage have lagged behind those for other major causes of child deaths. These need to be reassessed in the light of changing clinical guidelines and new efforts to promote community case management of pneumonia. **Two urgent challenges are: (1) to identify the best methods for assessing short-term trends in these indicators, so that the results can be used to improve program approaches and delivery strategies; and (2) to identify methods for assessing and reporting uncertainty.**

**Demographic and Health Surveys (DHS) and Multiple Indicator Cluster Surveys (MICS)**

DHS and MICS are household surveys designed to assess coverage levels which are widely used in developing countries. To gain information about childhood morbidity and treatment practices (in addition to many other health-related data), the primary caregivers of the household are interviewed about their child’s recent health. The interview follows a structured questionnaire in which the mother is questioned about the presence or absence of specific signs and symptoms displayed during their child’s recent illness. The accuracy and reliability of these data has been questioned, in particular in relation to data on childhood pneumonia [Lanata 1994; Harrison 1995; Colham 2000].

Until about 2006, these surveys did not include an indicator on pneumonia treatment – instead a proxy was used based on caregivers’ reports of careseeking for children with cough and fast breathing who did not have a blocked or runny nose. The Phase 5 DHS protocol asks caregivers of children with a cough and fast or difficult breathing (but without a blocked or runny nose) who report giving the child a treatment to identify when treatment was started (days after illness began illness) and an open-ended question about what drugs were given, with prompting (“Any other drugs?”). Surveyors check boxes for “pill/syrup” or “injection” if an antibiotic is reported by the caregiver. We are not aware of any studies that have attempted to assess the validity of responses offered by caregivers to these questions.

There are two major obstacles to overcome in gaining accurate morbidity information about childhood pneumonia from maternal interview. The first problem is that the interview only provides the most likely diagnosis from the recalled signs. Therefore, diseases which have a very distinct set of clinical signs which are easily recognized by the caregiver (such as diarrhoea) are more easily and accurately diagnosed by these methods than those (such as pneumonia) which are more difficult to recognize by caregivers. Secondly, as with all health interview data, there is a significant problem with the recall period. A study conducted in Egypt found that the specificity of many terms fell over time. The choice of recall period for these surveys is a trade-off since it needs to be long enough to include sufficient illness episodes whilst short enough to minimize memory decay. A study to validate the pneumonia algorithm contained within the MEASURE DHS phase III (donated as just “DHS” in this protocol) and MICS surveys has not yet been conducted.

**Study rationale**

It has been proposed that the use of data from Demographic and Health Surveys (DHS) and Multiple Indicator Surveys (MICS) be used to assess the coverage of interventions such as antibiotic treatment of children with pneumonia and so monitor progress made in improving global coverage (MICS is only be administered to children brought for care at the study health facility). It is, therefore, essential that results from these surveys are reliable and accurate. A key current requirement is therefore to measure the validity of the indicator of coverage of pneumonia treatment with a recommended antibiotic (proportion of children aged 2 months – 5 years with pneumonia who receive a recommended antibiotic) as measured by DHS and MICS methods and to assess the performance of novel alternate measurement strategies.

No studies to validate the pneumonia algorithm contained within the current DHS and MICS surveys and to evaluate differing recall periods have yet been conducted. We therefore propose to conduct a validation study of the Demographic and Health Survey (DHS) and MICS data from questions related to antibiotic treatment of pneumonia. In this the classification of “suspected pneumonia” from the DHS protocol will be compared to the diagnosis of pneumonia made trained health facility staff using a standard clinical protocol. This study should provide a measurement of the extent of misclassification and this will facilitate the correct interpretation of these data. The study will also evaluate two alternate strategies to improve the validity of caregivers’ responses and assess whether either of these lead to an improved performance and are likely to be feasible to implement in large scale household surveys.

**Project overall aim**

The objective this project is to improve the measurement of reported antibiotic treatment for pneumonia in children 0 to 5 years of age by mothers.

**Project research questions**

- What is the sensitivity and specificity of the MEASURE DHS PHASE III and MICS questionnaires for caregiver recognition of pneumonia (compared to an expert physician gold standard diagnosis)?
- What are the equivalent estimates of sensitivity and specificity when using an amended survey tool which incorporates:
  - a pneumonia score?
  - video illustrations of signs of pneumonia presented to caregivers?
- What is the sensitivity and specificity of the MEASURE DHS PHASE III and MICS questionnaires for caregiver recall of antibiotic treatment in children with and without pneumonia? (compared to an accurate record of treatment made at time of prescription)?
- What are the equivalent estimates of sensitivity and specificity when using an amended survey tool which incorporates:
  - A paper-based “pill/medicine board” illustrating different locally available treatment options
  - A computer based “pill/medicine board” illustrating different locally available treatment options
- Is there a significant difference in the accuracy of caregiver recall of diagnosis or antibiotic treatment between the 2 weeks and 4 weeks follow up protocol?

**Proposed alternate approaches**

- a pneumonia score, with 10- 20 questions relating to signs and symptoms of pneumonia (see Annex – final version to be informed by local pilot).
- a video to demonstrate a child with signs and symptoms of pneumonia or of “cough or cold” to aid the accurate recall of the caregiver.
- pictures of locally available drugs/injections/syrups to aid the caregiver’s accurate recall of the treatment their child received.

**Study Outline**

Selection of Study Sites

Two sites have been selected: one urban site in Pakistan and one rural site in Bangladesh. Criteria for site selection included: 1) high rates of childhood pneumonia; 2) feasibility for mounting the study within a short time and at a high level of quality, based on past performance of the PI, capacity of the study team and reviews of facility records; and 3) cost (since funds to support this activity are limited).

A third site in sub-Saharan Africa would be highly desirable, and initial contacts have been made. Decisions about the Africa site will be made over the coming months based on whether a suitable site can be identified and the availability of project funds. In the meantime, the study will move forward in the two existing sites.

Characteristics of the sites are summarized in table 1.

**Table 1: Characteristics of the study sites**

| **Pakistan Urban** |  |
| --- | --- |
| *Geographic Location:* | Islamabad |
| *Principal Investigator:* | Professor T Hazir |
| *Subcontracting Institution:* | Children’s Hospital, Pakistan Institute of Medical Sciences, Islamabad, Pakistan. |
| *Strategy for recruitment of children with pneumonia:* | Pneumonia cases identified at PIMS, a major children’s hospital by a trained doctor |
|  |  |
| **Bangladesh Rural** |  |
| *Geographic Location:* | Four rural villages: Mirzapur, Gorai, Bhatgram, Jamurki |
| *Principal Investigator:* | Dr Shams El Arifeen |
| *Subcontracting Institution:* | ICDDR,B |
| *Strategy for recruitment of children with pneumonia:* | Pneumonia cases identified at local government health services by a trained doctor |

*Recruitment at clinic*

Children aged 0 to 5 years who present to a health clinic or hospital out-patient department suffering from acute respiratory symptoms will be eligible to be recruited into the study. The children will be assessed according to a standard protocol by a study doctor and key clinical and treatment details recorded. A diagnosis of pneumonia, severe pneumonia or of “cough or cold” (no pneumonia) following an agreed clinical classification system and details of all treatment prescribed will be carefully recorded. Children presenting primarily with an episode of recurrent wheeze (reactive airways disorder / asthma) or who have pneumonia severe enough to require hospital admission or who have symptoms of more than 4 weeks duration or who have already received treatment from health services for the current illness episode will be excluded (so as to limit recall bias associated with the admission).

A detailed address will be recorded and consent sought for a follow up visit. The study will aim to recruit and follow up successfully 300 children with pneumonia (or severe pneumonia if this is treated in the community) and 300 with “cough or cold” (no pneumonia) at each study site. An attempt will be made to recruit all children with pneumonia who present during the defined study period when a trained clinical researcher is available. Children with “cough or cold / no pneumonia” will be frequency matched to pneumonia cases so that in any one week the age and sex distribution of cases of pneumonia and “cough or cold” are similar.

All children will then be treated according to the standard management protocols of that particular clinic. It is expected that some of the study children with pneumonia will go on to develop severe pneumonia, and require hospital referral. If this should happen the local health worker will ensure that the child is referred to the nearest health facility to receive medical care. A detailed record of the treatment given to the caregiver will be recorded.

It is recognized that children attending a clinic with pneumonia could represent a biased group that are not fully representative of all sick children. We will collect DHS data on socio-economic status in order to compare the sample population to a wider population. Furthermore, it is possible that the consent process and information sheet may introduce a source of bias in the recording of recall of symptoms and events.

*Follow up at home*

The primary caregivers of these children will first be interviewed using the DHS and MICS questions relating to pneumonia (see Annex) either at 2 weeks (200 with pneumonia and 200 with cough or cold) or 4 weeks (100 with pneumonia and 100 with cough or cold) after diagnosis. This will follow the DHS and MICS procedures as closely as possible. The order of administering the DHS and MICS questionnaires will be alternated with each child so that each is administered first on 50% of occasions. After this is completed they will then also be questioned about specific signs of pneumonia (which will form a pneumonia score - see below) and will be asked to watch a video of a child suffering from pneumonia and of a child suffering from an upper respiratory infection (asking which best represents their child’ respiratory illness episode). Finally, a drug chart will then be used to show them pictures of locally used medicines with a request to identify which medicines, if any, their child was prescribed. These research workers should be given the address details of the child but not be made aware of the study group of the child nor have access to the clinical details of the child recorded at recruitment.

*Home visit interview sequence:* The research worker will

1. introduce him/herself to the caregiver, and check that the details match those on the recruitment form.
2. inform the mother of the interview process and ensure that she is willing to participate (and obtain written consent).
3. check that the child’s details are correct, and make sure that the caregiver understands that the interview only concerns this child, and that she should only answer the questions in relation to this child.
4. start by asking the DHS questions (Annex 1) then ask MICS questions (Annex 2) – this sequence will be alternated from child to child to that DHS is asked first in 50% of children
5. ask pneumonia score questions (Annex 3)
6. show the caregiver the video clips and ask mother to identify which disease best represents her child
7. show the caregiver a drugs chart and ask her to identify with what her child was treated; note whether treatment information is based on this or actual observation of medications in the home
8. ask if the mother has a pharmacist’s note or a ‘road to health’ card, and record any information from the past 2 or 4 weeks.

*Study analysis*

The study report will include a detailed description of the epidemiological and health services characteristics of the study population

There will be 4 separate groups interviewed in this study:

- Mother’s whose child has been accurately diagnosed as having pneumonia (or severe pneumonia if this is treated in the community) by a physician, and is interviewed after 2 weeks.
- Mother’s whose child has been accurately diagnosed as having pneumonia (or severe pneumonia if this is treated in the community) by a physician, and is interviewed after 4 weeks.
- Mother’s whose child has been accurately diagnosed as not having pneumonia by a physician, and is interviewed after 2 weeks
- Mother’s whose child has been accurately diagnosed as not having pneumonia by a physician, and is interviewed after 4 weeks

Data from the interviews and the clinical case study forms will be used to calculate sensitivities and specificities of each of the above methods (DHS / MICS defined pneumonia; pneumonia defined by various pneumonia score thresholds; video-assisted recognition of pneumonia) for the diagnosis of pneumonia and for antibiotic treatment. These will be estimated for all possible pneumonia score thresholds so that an optimum performance threshold can be defined for the score. The caregiver will then be asked to show the research worker any pharmacist’s note, ‘road to health’ card or other health record which may have details of the illness and treatment given.

*Sensitivity + specificity calculations:*

|  | | **Medical diagnosis of Pneumonia** | | |
| --- | --- | --- | --- | --- |
|  | | **+ve** | **-ve** | **Total** |
| **diagnosis of “suspected pneumonia”**  **from DHS*** | **+ve** | **a** | **B** | **a+b** |
|  | **-ve** | **c** | **D** | **c+d** |
|  | **Total** | **a+c** | **b+d** | **n** |

***This same table will be used for the MICS diagnosis**

|  | | **Medical diagnosis of Pneumonia** | | |
| --- | --- | --- | --- | --- |
|  | | **+ve** | **-ve** | **Total** |
| **Does child reach specific pneumonia score (e.g. 5)** | **Y** | **a** | **B** | **a+b** |
|  | **N** | **c** | **D** | **c+d** |
|  | **Total** | **a+c** | **b+d** | **n** |

|  | | **Medical diagnosis of Pneumonia** | | |
| --- | --- | --- | --- | --- |
|  | | **+ve** | **-ve** | **Total** |
| **diagnosis of pneumonia**  **from video** | **+ve** | **a** | **B** | **a+b** |
|  | **-ve** | **c** | **D** | **c+d** |
|  | **Total** | **a+c** | **b+d** | **n** |

- Sensitivity = a/(a+c)
- Specificity = d/(d+b)

We will use Receiver operating characteristic (ROC) curves to present the results of the pneumonia score to determine the most appropriate score threshold.

We will also assess the validity of the report of treatment received in the same way.

|  | | **Child received drugX*** | | |
| --- | --- | --- | --- | --- |
|  | | **Y** | **N** | **Total** |
| **Mother said child received drugX*** | **Y** | **a** | **B** | **a+b** |
|  | **N** | **c** | **D** | **c+d** |
|  | **Total** | **a+c** | **b+d** | **n** |

*Drug X will be substituted for each of the medications listed

**Sample size calculation (see Table 2)**

The sample size calculation was based on two main considerations:

- Sample size to estimate sensitivity and specificity with a precision of +/- 5% based on baseline estimates of sensitivity of 60-70% and specificity of 70-90% (see table). Table 2 suggests that a sample size of 300 cases and 300 controls will achieve this.
- Sample size to distinguish a 10% difference (considered to be a programmatically important difference) in sensitivity and specificity with 80% power between estimates based on 2 week versus 4 week recall. Depending on the baseline level of sensitivity and specificity Table 2 suggests that a sample size of approximately 300 cases and 300 controls will achieve this. Note that a combined analysis across the two sites should have power to detect a smaller difference of the order of +/- 7-8%

Each study will therefore aim to achieve a completed sample size (with successful follow up) of 300 children under age five with physician-confirmed clinical pneumonia, and 300 children confirmed by a physician not to have pneumonia.

**Table 2 Sample size estimates**

Hence each study should have a sample size of 600 (300 children with pneumonia and 300 children with “cough or cold”) on whom a follow up appointment is completed. The comparison of 2 versus 4 week recall will be based on a comparison of 300 (200 pneumonia + 200 non pneumonia) with 2 week follow up versus 300 (100 pneumonia + 100 non pneumonia) with 4 week follow up at each site. As well as presented individually for each site results will be summarized over all study sites.

**Study organization and management**

**Study Management**

Professor Campbell will have overall responsibility for the study. He will be assisted by Dr. Shamim Qazi, who will conduct initial visits to each site to finalize the study protocol and ensure data quality and comparability across sites, and a follow-up visit to each site shortly after data collection begins. Dr Shams el Arifeen and Professor Tabish Hazir will be responsible for managing the day-to-day activities and conducting the primary analysis.

*Site specific preparation before recruitment*

*Ethical approval*

This will be sought from the appropriate authorities at each study sites and WHO Ethics committee. Each site will be responsible for preparing study recruitment, follow up and consent forms in the local language and for obtaining local ethical approval.

*Producing a pneumonia video*

Each site will be required to produce a video (at least 2 minute clip) of at least one child displaying the clinical signs of pneumonia (fast breathing of at least 10 breaths/minute above the threshold for that age and lower chest indrawing ) and at least one child demonstrating signs of “cough or cold” (blocked or runny nose and noisy upper respiratory breathing but NO fast breathing at least 10 breaths /minute below the age threshold) and upload it onto the study PC (or DVD player) with a medium sized screen that will be used for the interviews. There should be a video made of a 2 month old child and a 2 year old child for both pneumonia and “cough and cold”. The pilot test of the video will check that the video quality is appropriate for the study so that poor quality does not give falsely poor results.

*Developing a pneumonia score*

The pilot study will include a check of the face validity and cultural appropriateness of the items in the study protocol together with some formative research to identify local and commonly used terms for some of the items in the list, e.g. restlessness, flared nostrils, irritability etc.

*Identifying and documenting local treatments*

Each study site will also be required to document all drugs which are commonly used in that area to treat pneumonia, malaria and other febrile illnesses. They will also be required to take pictures of the individual pills/syrups/injections, which will then either be loaded onto the study PC, or printed on to a “pill board”.

*Training*

An experienced senior researcher will visit each study site before and once during the study period to ensure study procedures are being followed closely and in a similar fashion at each site. Training of clinic doctors and research worker s who conduct the follow up will cover

- Diagnosis of pneumonia
- Preparation of local pneumonia score and videos
- Study record forms
- MICS or DHS survey procedures
- Pilot study using the study procedures to recruit and follow up children to check that there are no major flaws in the planning or structure of the study and to address any problems which arise (this should include at least 5 children with pneumonia and 5 with “cough or cold”.
- Technical issues related to the video / PCs

This study forms will only be finalised after the training and pilot period and with the active participation of Dr Shamim Qazi, WHO.

Plan of Analysis

The data sets will be used to describe the sensitivity and specificity of reported signs of suspected pneumonia for “proven pneumonia” (a pneumonia score system will give a range of sensitivities and specificities for each score – which will enable a cut off score to be selected with the best sensitivity and specificity) for the current DHS protocol and for alternative protocols using the combinations of the three strategies described above. In addition, the study team will compare the results obtained using two- and four-week recall periods.

Project outcomes:

- Sensitivity + specificity of interview based diagnosis of pneumonia with existing DHS / MICS survey methods; pneumonia score and video-assisted recall.
- Sensitivity + specificity of mothers recall of treatments with existing DHS / MICS survey methods and new pictorial approaches

Comparison of results from 2 weeks and 4 weeks recall period (including a comparison of recalled timing of delay in careseeking at these different time points).

**Experience of the study investigators**

**Principal Investigators**

Professor Harry Campbell and Dr Igor Rudan of the University of Edinburgh are co-principal investigators. They have provided leadership on the epidemiology of pneumonia to the Child Health Epidemiology Reference Group (CHERG) since its inception. They are also members of all existing technical groups working on pneumonia epidemiology and scaling-up efforts to reduce pneumonia deaths, such as the Global Action Programme on Pneumonia (GAPP) and the technical working groups on pneumonia within the CHERG. They have published >50 original articles on child health and childhood pneumonia.

**Investigators**

Dr. Shamim Qazi of the World Health Organization will coordinate establishment of study procedures and monitor adherence with study protocols and data quality at the study sites. He has had a leadership role in establishing global policies to tackle childhood pneumonia over the past 10 years and, in particular in the establishment of GAPP. He has extensive developing country experience in setting up and monitoring successful clinical studies on childhood pneumonia and has published more than 30 original articles on childhood pneumonia.

Dr Shams el Arifeen and Professor Tabish Hazir will be the local lead investigators responsible for the successful achievement of the study aims at the two study sites. They both have extensive experience of field research on childhood pneumonia in these locations and both have strong track records of achievement and publication in high impact journals such as the Lancet. They have published about 100 original articles in total on various aspects of child health, with a major focus within this on pneumonia.

The CHERG MA13 Core Group will stay abreast of study developments and offer technical inputs for consideration by the investigators.

**Time Line**

Results are expected by the end of 2010.

**NOTES**

*Pneumonia score:*

A set of 10 items from a list of 20 possible questions will be selected at each study site (based on what local doctors / researchers consider to be most culturally appropriate and based on the findings of the pilot study - selected by the local PI based on level of comprehension by caregivers, performance in comparison to known diagnoses and the informed opinion / experience of the local PI). This list represents symptoms and signs of pneumonia which are of increasing severity. The interviewer will ask the caregiver about the presence of each specific sign separately, and record her responses. The clinical signs are:

- Cough
- Fever
- Sweating
- Irritability
- Fatigue
- Restlessness
- Fast breathing
- Shortness of breath
- Loss of appetite
- Decreased water intake / drinking
- wheezing
- Chest pain
- Difficulty breathing
- Intercostal or subcostal retractions
- Flared nostrils
- Grunting
- Repeated vomiting
- Cyanosis
- Haemoptysis / blood when child coughs
- Fits

*Video clips*

During the interview, the research worker will show the caregiver a short video clip showing a child suffering from pneumonia, followed by a clip showing a child with a “cough or cold”. The videos will show clinical signs commonly associated with both, and the mother will be asked which clip most accurately shows the signs her child was displaying when they were ill. The research worker will record whether the caregiver selected the child with pneumonia, or the child with “cough or cold”.

- Child with signs of pneumonia: clinical signs of pneumonia (fast breathing of at least 10-15 breaths /minute above the threshold for that age and /or chest indrawing)
- Child with signs of “cough or cold”: (blocked or runny nose and noisy upper respiratory breathing but NO fast breathing at least 10 breaths /minute below the age threshold)

There should be a video made of a 2 month old child and a 2 year old child for both pneumonia and “cough and cold”. An alternative to be explored in the pilot study is to make separate videos of children with “cough and cold”, pneumonia (fast breathing) and severe pneumonia (chest indrawing). The research worker should select the age category that will best represent the child which is being recruited and show the relevant clips to the mother.

*Drug Chart*

During the interview, the research worker will show the caregiver a chart which will contain pictures of all the locally used medications. The research worker will ask the caregiver which, if any, of these treatments her child received. This drug chart should be presented both as a separate laminated sheet and loaded onto the study laptop PC to be displayed after the video is shown. The drugs shown should be clearly displayed, with any identifying marks (e.g. markings) easily visible. The caregiver will also be asked about details of dose and duration of treatment given and source of the medication. The pilot study will consider methods proposed in the WHO manual edited by Hardon before the final study protocol is established.

**Annexes:**

**Annex 1: DHS(V) questions – these will be replaced by the MEASURE DHS phase III questions (in related document “Copy of DHS6 Woman's QRE 16 March pneumonia-1”**

533 *Has (NAME) been ill with a fever at any time in the last 2 weeks?*

*Yes 1*

*No 2*

*Don’t Know 8*

1. *Has (NAME) had an illness with a cough at any time in the last 2 weeks?*

*Yes 1*

*No 2*

*Don’t Know 8*

1. *When (NAME) had an illness with a cough, did he/she breathe faster than usual with short, rapid breaths or have difficulty breathing?*

*Yes 1*

*No 2*

*Don’t Know 8*

1. *Was the fast or difficult breathing due to a problem in the chest or to a blocked or runny nose?*

*Chest Only 1*

*Nose Only 2*

*Both 8*

*Other _______________ (Specify) 6*

*Don’t Know 8*

1. *Check 533: Had Fever?*
2. *Now I would like to know how much (NAME) was given to drink (including breastmilk) during the illness with a (fever/cough). Was he/she given less than usual to drink, about the same amount, more than usual to drink?*

*If less, probe: Was he/she given much less than usual to drink or somewhat less?*

*Much Less 1*

*Somewhat Less 2*

*About the Same 3*

*More 4*

*Nothing to Drink 5*

*Don’t Know 6*

1. *When (NAME) had a (fever/cough), was he/she given less than usual to eat, about the same amount, more than usual, or nothing to eat?*

*If less, probe: Was he/she given much less than usual to eat or somewhat less?*

*Much Less 1*

*Somewhat Less 2*

*About the Same 3*

*More 4*

*Stopped Food 5*

*Never Gave Food 6*

*Don’t Know 6*

1. *Did you seek advice or treatment for the illness from any source?*

*Yes 1*

*No 2*

1. *Where did you seek advice or treatment? Anywhere else?*

*Probe to identify each type of source and circle the appropriate code(s).*

*If unable to determine if a hospital, health center or clinic is public or private medical, write the name of the place.*

*(Name of place)*

*Public Sector*

*Govt Hospital A*

*Govt Health Center B*

*Govt Health Post C*

*Mobile Clinic D*

*Fieldworker E*

*Other Public F*

*________________ (specify)*

*Private Medical Sector*

*PVT Hospital/Clinic G*

*Pharmacy H*

*PVT Doctor I*

*Mobile Clinic J*

*Fieldworker K*

*Other Private Med. L*

*________________ (specify)*

*Other Source*

*Shop M*

*Traditional Practitioner N*

*Other X*

*________________ (specify)*

1. *Were did you first seek treatment? Use letter code from 541.*
2. *How many days after the illness began did you first seek advice or treatment for (NAME)? If the same day, record ‘00’.*
3. *Is (NAME) still sick with (fever/cough)?*

*Fever Only 1*

*Cough Only 2*

*Both Fever and Cough 3*

*No, Neither 4*

*Don’t Know 5*

1. *At any time during the illness, did (NAME) take any drugs for the illness?*

*Yes 1*

*No 2*

1. *What drugs did (NAME) take? Any other drugs? Record all mentioned.*

*Antimalarial Drugs*

*SP/Fansidar A*

*Chloroquine B*

*Amodiaquine C*

*Quinine D*

*Combination with Artemisinin E*

*Country Spec. CBD Antimalarial F*

*Other Antimalarial G*

*________________ (specify)*

*Antibiotic Drugs*

*Pill/Syrup H*

*Injection I*

*Other Drugs*

*Aspirin J*

*Acetaminophen K*

*Ibuprofen L*

*Other ________________ (specify) X*

*Don’t Know Z*

*Followed by specific questions about each drug mentioned…*

**Annex 2: MICS questions**

| CA7. At any time in the last two weeks, has (name) had an illness with a cough? | Yes 1  No 2  DK 8 | 2⇨CA14  8⇨CA14 |
| --- | --- | --- |
| CA8. When (name) had an illness with a cough, did he/she breathe faster than usual with short, rapid breaths or have difficulty breathing? | Yes 1  No 2  DK 8 | 2⇨CA14  8⇨CA14 |
| CA9. Was the fast or difficult breathing due to a problem in the chest or a blocked or runny nose? | Problem in chest 1  Blocked or runny nose 2  Both 3  Other (specify) 6  DK 8 | 2⇨CA14  6⇨CA14 |
| CA10. Did you seek any advice or treatment for the illness from any source? | Yes 1  No 2  DK 8 | 2⇨CA12  8⇨CA12 |
| CA11. FROM WHERE DID YOU SEEK ADVICE OR TREATMENT? |  |  |
| CA12. Was (name) given any medicine to treat this illness? | Yes 1  No 2  DK 8 | 2⇨CA14  8⇨CA14 |
| CA13. What medicine was (name) given?  Probe:  Any other medicine?  Circle all medicines given. Write brand name(s) of all medicines mentioned.    (Names of medicines) | Antibiotic  Pill / Syrup A  Injection B  Anti-malarials M  Paracetamol / Panadol / Acetaminophen P  Aspirin Q  Ibuprofen R  Other (specify) X  DK Z |  |

**Annex 3:** **Pneumonia score questions**

*1 Has (name) suffered from a* ***cough*** *in the past (___) weeks?*

*2 Has (name) had a* ***fever*** *in the past (___) weeks?*

*3 Has (name) suffered form* ***chills or sweats*** *over the past (___) weeks?*

*4 Has (name) been* ***restless*** *over the past (___) weeks?*

*5 Has (name) been unusually* ***irritable*** *over the past (___) weeks?*

*6 Has (name) had any* ***loss of appetite*** *over the past (___) weeks?*

*7 Has (name) been* ***abnormally sleepy*** *or* ***difficult to wake*** *over the past (___) weeks?*

*8 Have you noticed (name) making a* ***wheezing*** *sound when he/she breathed over the last (__) weeks?*

*9 Has (name) appeared* ***short of breath*** *over the past (___) weeks?*

*10 Has there been a period over the past (___) weeks when (name) has seemed to be* ***breathing faster*** *than normal?*

*11 Have you noticed (name’s)* ***nostrils flaring*** *when he/she breathed over the past (___) weeks?*

*12 Has (name)* ***refused to drink*** *or had a definite* ***decrease in the amount of water he / she drank*** *over the past (___) weeks?*

*13 Did you notice that (name’s)* ***skin between their ribs, or his/ her tummy move inwards*** *when they breathed over the past (___) weeks*

*14 Did (name) seem to have any* ***chest pain*** *over the past (___) weeks?*

*15 Has there been a period over the last (___) weeks where (name) seemed to have* ***difficulty breathing****?*

*16 Has (name)* ***vomited*** *at all over the past (___) weeks?*

*17 Have you heard (name) making* ***grunting*** *noises during the past (___) weeks?*

*18 Has (name) appeared to have a slightly* ***blue coloration*** *to his/her skin, particularly their lips or tongue, over the past (___) weeks*

*19 Has (name)* ***coughed up any blood*** *over the past (___) weeks?*

*20 Has (name) had any* ***fits*** *overt the past (___) weeks*

**Annex 4:** **DRAFT** **Form to be completed by Physician**

Section 1: patient details

Child’s Name (full): ………………………………………........................................

Age: …………………………………………………………………………………...

D.O.B: ………………………………………………………………………………...

Mother’s/

primary caregiver’s name: ………………………………………………......................

Address: ……………………..................

……………………..................

……………………..................

……………………..................

……………………..................

Date of examination:………………………………………………………………....

Section 2: presenting signs + symptoms

Respiratory symptoms: ……………………………………………………………...

……………………………………………………………..

……………………………………………………………..

Respiratory rate:

Temperature (^o^C):

Sub-costal retractions / lower chest wall indrawing (y/n):

Record any findings on auscultation -

Section 3: Investigation

Chest X-ray ordered (y/n):

Section 4: Diagnosis

Pneumonia non-pneumonia (circle appropriate)

Presence of exclusion criteria

- - recurrent wheezing
  - chronic cough (more than 10 days)
  - already on antibiotic treatment
  - recent pneumonia episode within past 10 days
  - history of congenital heart disease
  - symptoms for more than 4 weeks
  - previous clinic treatment for this illness episode

Section 5: Treatment (to be adapted for each site)

| *Drugs* | | *Please tick those prescribed* |
| --- | --- | --- |
| *Antimalarial Drugs* | *SP/Fansidar* |  |
|  | *Chloroquine* |  |
|  | *Amodiaquine* |  |
|  | *Quinine* |  |
|  | *Combination with Artemisinin* |  |
|  | *Country Spec. CBD Antimalarial* |  |
|  | *Other Antimalarial* | **Specify:** |
|  |  |  |
| Antibiotic Drugs | *Pill/Syrup* |  |
|  | *Injection* |  |
|  |  |  |
| Other Drugs | *Aspirin* |  |
|  | *Acetaminophen* |  |
|  | *Ibuprofen* |  |
|  | *Other* | **Specify:** |

Physician name:………………………………..

Date:……………………………………………

**Annex 5:** **Home follow up form**

Child’s Name (full): ………………………………………........................................

Age: …………………………………………………………………………………...

D.O.B: ………………………………………………………………………………...

Mother’s/

primary caregiver’s name: ………………………………………………......................

Address: ……………………..................

……………………..................

……………………..................

……………………..................

……………………..................

Date interviewed:………………………………………………………………...

DHS / MICS questions – TO BE ADDED

Pneumonia score – TO BE ADDED

Video exercise findings – TO BE ADDED

Treatment received – TO BE ADDED

**ANNEX 6:** **INFORMED CONSENT FORM**

**Informed Consent Form for:** Health interview of the caregivers of children (1month-5years of age) who present to health clinic with respiratory symptoms.

**Title of Research Project:** Validation of caregiver report of childhood pneumonia and antibiotic treatment from Demographic and Health Survey (DHS) data and assessment of alternative approaches.

**Local lead investigators: Prof Tabish Hazir (Pakistan); Dr Shams el Arifeen (Bangladesh)**

**Organisation: Children’s Hospital, Islamabad and ICDDR,B**

**Sponsor: University of Edinburgh**

**Protocol Number:**

**PART 1: Information**

***(Instructions to Physician: Read this statement and explain the consent to the to the mother of the child, and answer any questions she may have.)***

**Introduction and Purpose of the Research:**

Health surveys where mothers and other caregivers are asked about the health of their child or about a recent illness episode are carried out by the government in this country. This is done to give information to help improve child health programmes. We are research project trying to improve the quality of information gained from surveys about the treatment of childhood pneumonia.

**Why selected:**

Since you live in our study area, and your child is between the ages of 1 month and 5 years, we would like to ask you to be involved in our study.

**What is expected of the Respondent:**

If you agree to take part, in 2 or 4 weeks time a field worker will visit your house and ask you questions about the recent health of your child. The visit will last no longer than 20 minutes. We will then compare your answers with your child’s medical records, to see how accurate the information we were able to gain from you was. You will be shown a short video clip and photograph and asked some questions.

**Risks and Benefits:**

There is no risk posed to either you or your child because of our study. If any questions make you feel uncomfortable during the interview you do not have to answer them.

There will be no direct or immediate benefit to you or your child from participating in this study. However, the results of this study will help to assess how accurate the pneumonia information from household surveys is. This information could be sued to improve childhood pneumonia programmes and so help improve the health of the children in your community.

**Privacy, anonymity and confidentiality:**

Privacy, anonymity and confidentiality of any data/information identifying you will be strictly maintained. We will keep all information you provide us with private and make sure it is secure. Members of our research team will do the processing of the data in a secure place. Only people involved in this research will have normally have access to this information. Any data that we send to other research groups will not include any of your personal identifiable information.

Please feel free to ask any questions. We will be happy to answer them for you. You can also contact any members of our research team (contact address provided) if you have nay questions at a later date. Also, once the study is completed the results will be available for you to see if you are interested.

**Future use of Information:**

The results of our study may be shared with other health or health research organisations, but none of your personal information or your names will ever be shared with anyone. We will always uphold your privacy, anonymity and confidentiality.

**Right not to participate and withdraw:**

You do not need to agree to take part in this study. You alone have the sole choice of whether you wish to take part or not. There will be no difference in the care provided for your child, whether you agree to take part or not. If you do decide to take part, you are free to withdraw at any stage. Also, during the interview, if there are any questions that you do not want to answer then please feel under no obligation to do so.

**Principle of compensation:**

Your participation in this study is completely voluntary, and you will not be paid for taking part.

If you agree to our proposal, and are willing to take part in our research please indicate this by putting your signature, or left thumbprint impression in the specified place below.

**PART 2: Certificate of consent**

***(Instructions to Physician: Read this statement and explain the consent to the to the mother of the child, and answer any questions she may have.)***

**I have been asked to give my consent for my child and to be involved in this study, which will involve me being interviewed about my child’s recent health. I have been read the information above and understand fully what the study will involve. I have had the opportunity to ask any questions that I have wanted, and any questions that I have asked have been answered to my satisfaction. I voluntarily consent to myself and my child being involved in this study.**

**If illiterate:** *a witness must be present and the mother should give her thumb print. (The witness should be selected by the mother if possible and not be an member of the research team.)*

**Print name of Mother…………………………………**

**Signature/**

**left thumbprint of Mother…………………………………...**

**Date…………………………………………………….**

Day/Month/Year

**Print name of Witness…………………………………**

**Signature/**

**left thumbprint of Witness…………………………………..**

**Date…………………………………………………….**

Day/Month/Year

**Statement by the research person taking consent:**

**I have read out all of this information to the mother and made sure to the best of my ability that she understands that the following will be done:**

1. **A field worker will interview her 2 or 4 weeks after the child was brought to the clinic.**
2. **The interview will concern her child’s recent health and treatment**
3. **She is under no obligation to take part in this study, or answer any questions during the interview that she is not comfortable in doing so.**
4. **All information about her and her child will remain confidential.**

**I confirm that the mother was given opportunity to ask any questions that she may have had, and that I answered them all correctly and to the best of my ability. I confirm that the mother has not been coerced into giving consent, and that it has been given freely and voluntarily.**

**A copy of this consent form has been given to the mother.**

**Print name of researcher/**

**person taking consent……………………………………..**

**Signature of researcher/**

**person taking consent……………………………………….**

**Date…………………………………………………….**

Day/Month/Year

References

Black RE, Cousens S, Johnson H, Lawn J, Rudan I, Bassani D, Pha J, Campbell H, Fischer Walker C, Cibulskis R, Eisele T, Liu L, Mathers C for the Child Health Epidemiology Reference Group of the World Health Organization and UNICEF

Global, Regional and National Causes of Child Mortality, 2008. Lancet 2010: in press

Colham et al 2000. Prospective validation of a standardised questionnaire for estimating childhood mortality and morbidity due to pneumonia and diarrhoea.

Harrison et al 1995. Maternal reporting of Acute Respiratory Infection in Egypt..

Lanata et al 1994. Validity of a Respiratory Questionnaire to identify pneumonia in children in Lima, Peru.

Kalter et al. 1999. Validation of caregiver interviews to diagnose common causes of severe neonatal illness.

Rudan I, Tomaskovic L, Boschi-Pinto C, Campbell H; WHO Child Health Epidemiology Reference Group. Global estimate of the incidence of clinical pneumonia among children under five years of age. Bull World Health Organ. 2004; 82: 895-903.

Rudan I, Boschi-Pinto C, Biloglav Z, Mulholland K, Campbell H. Epidemiology and etiology of childhood pneumonia. Bull World Health Organ. 2008; 86: 408-16.

WHO. *World Health Statistics 2007*. Geneva, World Health Organization, 2007. Available from: <http://www.who.int/whosis/whostat2007.pdf>
